# Design and synthesis of a potent, highly selective, orally bioavailable, retinoic acid receptor alpha agonist

**DOI:** 10.1016/j.bmc.2017.12.015

**Published:** 2018-02-15

**Authors:** Earl Clarke, Christopher I. Jarvis, Maria B. Goncalves, S. Barret Kalindjian, David R. Adams, Jane T. Brown, Jason J. Shiers, David M.A. Taddei, Elodie Ravier, Stephanie Barlow, Iain Miller, Vanessa Smith, Alan D. Borthwick, Jonathan P.T. Corcoran

**Affiliations:** a Neuroscience Drug Discovery Unit, Wolfson Centre for Age-Related Diseases, Guy’s Campus, King’s College, London SE1 1UL, UK; b DrugMolDesign, 15 Temple Grove, London NW11 7UA, UK; c Sygnature Discovery Limited, Biocity, Pennyfoot Street, Nottingham NG1 1GF, UK

## Abstract

A ligand-based virtual screening exercise examining likely bioactive conformations of AM 580 (**2**) and AGN 193836 (**3**) was used to identify the novel, less lipophilic RARα agonist 4-(3,5-dichloro-4-ethoxybenzamido)benzoic acid **5**, which has good selectivity over the RARβ, and RARγ receptors. Analysis of the medicinal chemistry parameters of the 3,5-substituents of derivatives of template **5** enabled us to design a class of drug-like molecules with lower intrinsic clearance and higher oral bioavailability which led to the novel RARα agonist 4-(3-chloro-4-ethoxy-5-isopropoxybenzamido)-2-methylbenzoic acid **56** that has high RARα potency and excellent selectivity versus RARβ (2 orders of magnitude) and RARγ (4 orders of magnitude) at both the human and mouse RAR receptors with improved drug-like properties. This RARα specific agonist **56** has high oral bioavailability (>80%) in both mice and dogs with a good PK profile and was shown to be inactive in cytotoxicity and genotoxicity screens.

## Graphical abstract

**Figure:**
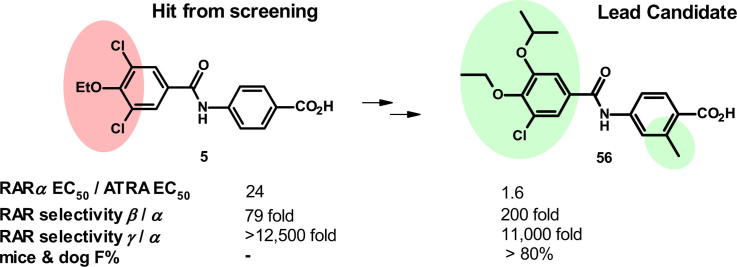

## 1 Introduction

The retinoic acid receptors (RARα, RARβ, and RARγ) are members of the nuclear receptor superfamily. Compounds which bind to and activate the RARs are termed retinoids and comprise both natural retinol (Vitamin A) metabolites and synthetic analogs. Retinoids regulate a wide variety of biological processes such as vertebrate embryonic morphogenesis and organogenesis, cell growth arrest, differentiation, and apoptosis, as well as their disorders.^1^

The RARα isoform is found in the majority of tissues and has been implicated in a number of diseases, most notably acute promyelocytic leukemia (APL). Selective RARα agonists have been shown to inhibit proliferation and induce apoptosis of mammary tumor oncogenesis in murine models (MMTV-neu and MMTV-wnt1 transgenic mice) relevant to human cancer,^2^ and to inhibit LPS-induced B-lymphocyte proliferation.^3^ Selective RARα agonists have also been shown to prevent neuronal cell death caused by amyloid-β and, when administered orally, can prevent amyloid-β production and Alzheimer’s disease progression in a mouse model.^4^ It has been shown^5^ that selective RARα agonists suppressed allospecific immune response and significantly prolonged the survival of mouse cardiac allografts and can ameliorate nephritis in lupus-prone mice, NZB/NZW F1.^6^ This supports the rationale for using RARα agonists as immunosuppressants in human organ transplantation. Thus selective RARα agonists have the therapeutic potential for the treatment of cancer, dermatological diseases, Alzheimer’s disease and immunological disorders.

**Fig. 1:**
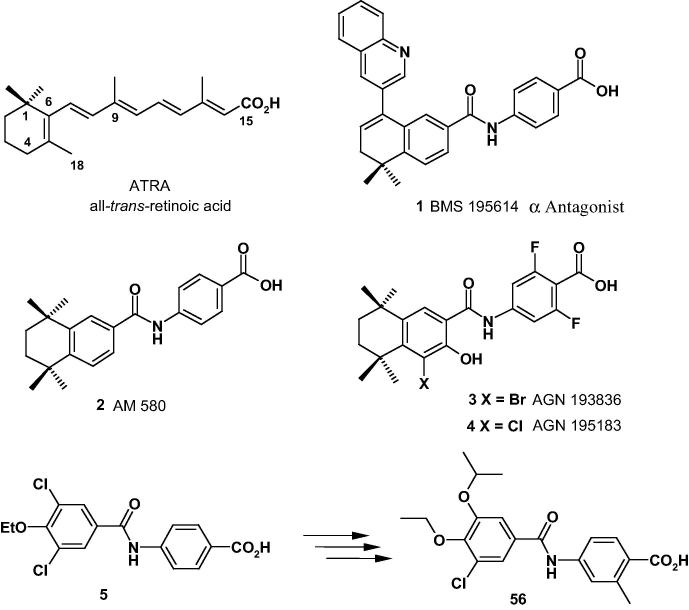
RARα agonists and antagonist.

Synthetic RARα, RARβ, and RARγ agonists have been developed from all-*trans*-retinoic acid (ATRA), and usually consist of a lipophilic ring, a linker and a carboxylic acid (Fig. 1). There has been an extensive studies on the SAR^7^, ^8^, ^9^, ^10^ of the RARα agonists based essentially on the bicyclic 5,5,8,8-tetramethyl-5,6,7,8-tetrahydronaphthalene derivatives which evolved from ATRA. All three agonist, AM 580 (**2**), AGN 193836 (**3**), AGN 195183 (**4**) and antagonist BMS 195614 (**1**) (Fig. 1) which contain an amide linker and a benzoic acid, arose from these studies.

However, although AM 580 (**2**) and AGN 195183 (**4**) have moderate and good selectivity respectively for RARα, over RARβ and RARγ they are quite lipophilic (cLog P 6.3 and 7.2). In addition AM 580 (**2**) has been shown to be toxic,^11^, ^12^ and the more recently discovered compound AGN 195183 (**4**)^10^ which was in Phase I clinical trials for cancer has been discontinued.^13^ Our aim was to find a novel, potent, highly selective RARα agonist not based on the bicyclic 5,5,8,8-tetramethyl-5,6,7,8-tetrahydronaphthalene class that was ligand efficient, orally bioavailable and without the lipophilic obesity seen with (**2**), (**3**) and (**4**). We outline here how we discovered our initial hit compound **5** and how this was developed into the orally bioavailable, highly potent and selective RARα agonist **56** (Fig. 1) which exhibits promising drug-like properties.

## 2 Chemistry

**Scheme 1:**
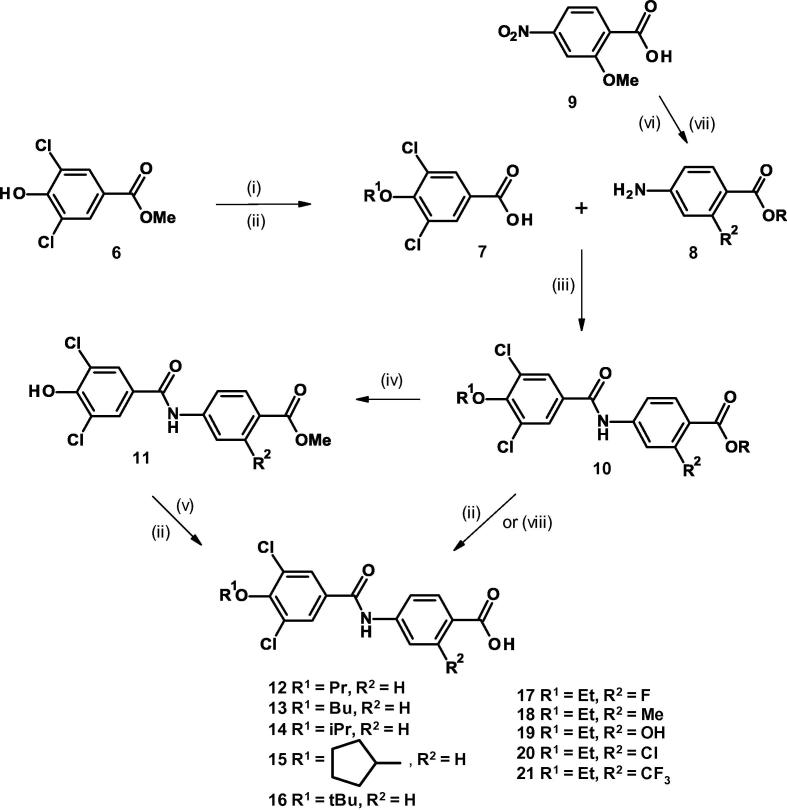
4-(3,5-Dichloro-4-alkoxy-benzamido)benzoic acids. (Reagents and conditions: (i) K_2_CO_3_, R^1^Br, DMF, 80 °C, 3 h; (ii) LiOH, THF, H_2_O, room temp, 12 h; (iii) (COCl)_2_,CH_2_Cl_2_, DMF, 0 °C, 1 h then methyl 4-amino-2-R^2^-benzoate, NEt_3_, room temp, 12 h or HATU, DMF, DIPEA, 5 min, then methyl 4-amino-2-R^2^-benzoate, DMF, room temp, 18 h; (iv) R^1^ = Bn, R^2^ = Me; BCl_3_, CH_2_Cl_2_, 0 °C then room temp, 12 h; (v) 1,1-di-*tert*-butoxy-*N,N*-dimethylmethanamine, toluene, 80 °C, 3 h, then room temp, 12 h; (vi) 1,1-di-*tert*-butoxy-*N,N*-dimethylmethanamine, toluene, 80 °C, 3 h, then further 1,1-di-*tert*-butoxy-*N,N*-dimethylmethanamine, 2 mol, added, 80 °C, 16 h. (vii) H_2_, Pd/C, MeOH, room temp; (viii) R^1^ = Et, R^2^ = MeO, R = *^t^*Bu; BCl_3_, CH_2_Cl_2_, 0 °C then room temp 2 h).

**Scheme 2:**
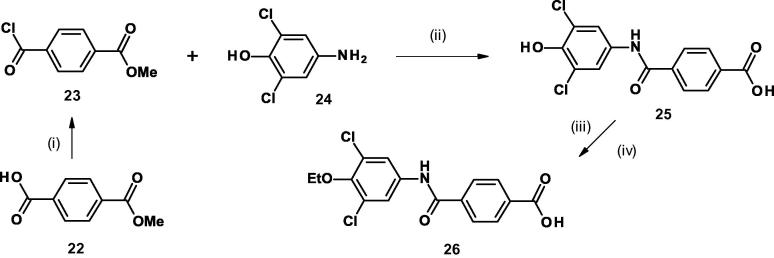
4-(3,5-Dichloro-4-ethoxyphenylcarbamoyl)benzoic acid. (Reagents and conditions: (i) (COCl)_2_,CH_2_Cl_2_, DMF, 0 °C, then room temp 2 h; (ii) DIPEA, CH_2_Cl_2_, room temp, 16 h, then LiOH, THF, H_2_O, room temp, 16 h; (iii) K_2_CO_3_, EtI, DMF, 65 °C, 18 h, then further EtI, 70 °C, 3 h; (iv) LiOH, THF, H_2_O, room temp, 5 h).

**Scheme 3:**
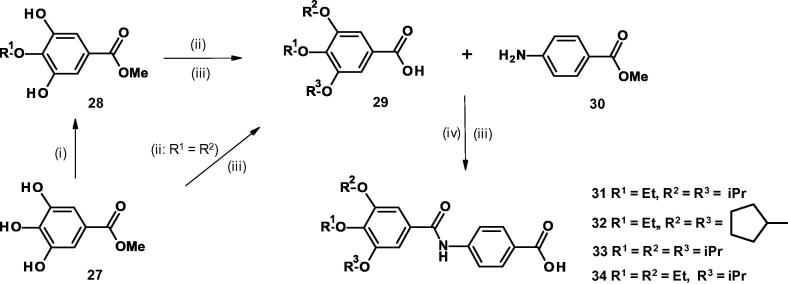
4-(3,4,5-Trialkoxybenzamido)benzoic acids. (Reagents and conditions: (i) NaHCO_3_, R^1^I, DMF, 30 °C, 72 h; (ii) K_2_CO_3_, R^2^Br, DMF, 50 °C, 48 h; (iii) LiOH, THF, H_2_O, room temp, 16 h; (iv) (COCl)_2_, CH_2_Cl_2_, DMF, 0 °C, 1 h then methyl 4-amino-benzoate, NEt_3_, room temp, 12 h).

**Scheme 4:**
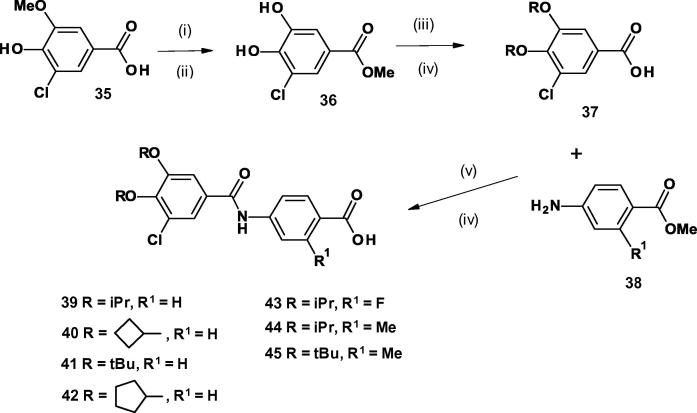
4-(3-Chloro-4,5-dialkoxybenzamido)benzoic acids with identical alkoxy groups. (Reagents and conditions: (i) BBr_3_, CH_2_Cl_2_, 0 °C, 2 h; (ii) TMSCl, MeOH, 50 °C, 16 h; (iii) K_2_CO_3_, RI, DMF, 70 °C, 46 h; (iv) LiOH, THF, H_2_O, room temp, 18 h; (v) (COCl)_2_, CH_2_Cl_2_, DMF, 0 °C, 1 h then methyl 4-amino-2-R^1^-benzoate, NEt_3_, room temp, 12 h).

**Scheme 5:**
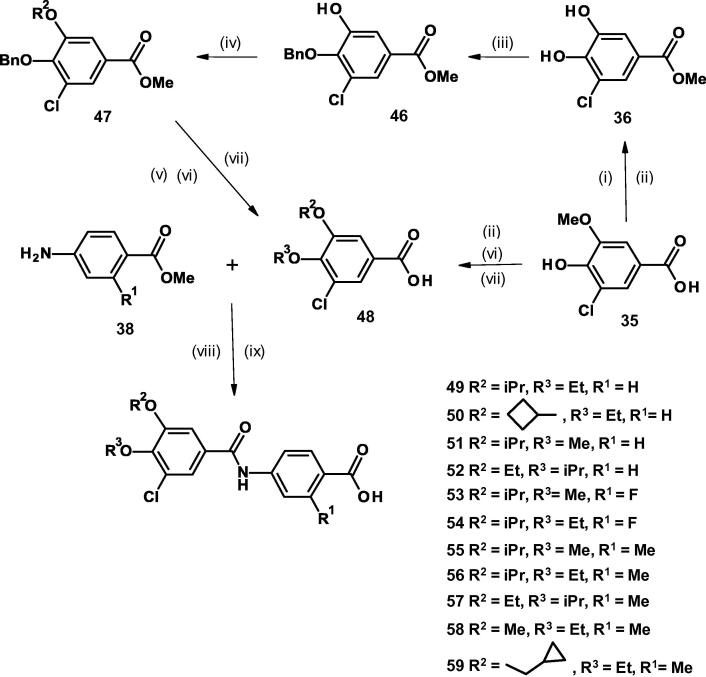
4-(3-Chloro-4,5-dialkoxybenzamido)benzoic acids with non-identical alkoxy groups. (Reagents and conditions: (i) BBr_3_, CH_2_Cl_2_, 0 °C, 2 h; (ii) TMSCl, MeOH, 50 °C, 16 h; (iii) K_2_CO_3_, BnBr, DMF, 60 °C, 0.75 h; (iv) K_2_CO_3_, R^2^Br, DMF, 60 °C, 2 h; (v) H_2_, 10% Pd/C, MeOH; (vi) K_2_CO_3_, DMF, 60 °C, 10 min, then R^3^I, 40 °C, 3 h; (vii) LiOH, THF, H_2_O, 40 °C, 1 h, then room temp, 16 h; (viii) T3P, methyl 4-amino-2-R^1^-benzoate, NEt_3_, EtOAc, 60 °C, 4 h, then room temp, 16 h. (ix) LiOH, THF, H_2_O, 40 °C, 16 h).

The phenyl carboxamido-benzoic acids (Scheme 1, Scheme 3, Scheme 4, Scheme 5) and phenyl carbamoyl-benzoic acid **26** (Scheme 2) were prepared by coupling the appropriately substituted aniline with a substituted benzoic acid using a variety of standard methods for the formation of an amide bond. The 3,5-dichloro-4-alkoxy compounds **12**–**15** and **17**–**21** (Scheme 1) were prepared by alkylation of the phenolic group of methyl 3,5-dichloro-4-hydroxybenzoate **6** followed by hydrolysis of the benzoate ester. Coupling the resultant acid **7** via the acid chloride by reaction with oxalyl chloride or directly with HATU, with the appropriate methyl 4-aminobenzoate **8** followed by hydrolysis with lithium hydroxide gave the required acids **12**–**15** and **17**–**21**.

For compound **16** the initial alkylation of **6** was carried out with benzyl bromide, and the resulting benzyloxy compound was hydrolyzed, coupled with the aniline **8** (R^2^ = H) and the benzyl group was removed using boron trichloride to result in compound **11** (R^2^ = H). This material was then alkylated using 1,1-di-*tert*-butoxy-*N,N*-dimethylmethanamine in toluene at 80 °C. A final hydrolysis using lithium hydroxide in a mixture of tetrahydrofuran and water gave the tertiary butoxy compound **16**.

A similar sequence (Scheme 2) coupling the aniline **24** and acid chloride **23 (**obtained from acid **22)** followed by hydrolysis gave the phenolic acid **25** which upon alkylation with ethyl iodide followed by hydrolysis gave the reverse amide analog **26**.

The 3,4,5-trialkoxybenzamido-benzoic acids **31**–**34** were prepared in four or five steps from methyl 3,4,5-trihydroxybenzoate **27** as illustrated in Scheme 3.

For the symmetrical tri-alkoxy compound **31**, treatment of **27** with sodium hydrogen carbonate and ethyl iodide gave mainly compound **28** (R^1^ = Et) where alkylation had only occurred in the 4-position of the substrate. After purification, this compound on treatment with potassium carbonate and 2-bromopropane gave an intermediate compound where both remaining hydroxyl groups had reacted with the alkylating reagent. Hydrolysis resulted in the fully alkylated benzoic acid **29** (R^1^ = Et, R^2^ = R^3^ = iPr) which was coupled via the acid chloride to give the methyl ester of compound **31**. A final hydrolysis using lithium hydroxide yielded compound **31**. The other tri-alkoxy derivatives **32**–**34** were similarly prepared (Scheme 3).

The 3-chloro-4,5-dialkoxybenzamido benzoic acids **39**–**45** and **49**–**59** were prepared as described in Scheme 4, Scheme 5. The commercially available 3-chloro-4-hydroxy-5-methoxybenzoic acid **35** was treated sequentially with boron tribromide and trimethylsilyl chloride in methanol to leave methyl 3,4-dihydroxy-5-chlorobenzoate **36**.

For the derivatives **39**, **40**, **42**, **43**, and **44**, (Scheme 4) where the alkoxy groups are the same, both hydroxyl groups in **36** were alkylated by using potassium carbonate and the appropriate alkyl halide in *N, N*-dimethylformamide heated to 70 °C.

Hydrolysis gave rise to the fully substituted benzoic acids **37 (**R = iPr**)**, **37** (R = cyclobutyl) and **37 (**R = cyclopentyl. These were then coupled to the aniline **38** via the acid chloride generated by treatment of the benzoic acid with oxalyl chloride.

The di-*tert*-butoxy derivatives **41** and **45** were synthesized from the acid **37 (**R = *^t^*Bu), which was prepared by reacting the two hydroxyl groups in **36** with *N, N*-dimethylformamide di-*tert*-butyl acetal followed by hydrolysis, and then coupling the product directly with aniline **38** using HATU. A final treatment of the coupled products with lithium hydroxide in aqueous 1,4-dioxane gave the required acids.

The non-identical di-alkoxy compounds **49**–**57** and **59** were also prepared via methyl 3,4-dihydroxy-5-chlorobenzoate **36**, while **58** was prepared from 3-chloro-4-hydroxy-5-methoxybenzoic acid **35** via benzoic acid **48** (R^2^ = Me, R^3^ = Et) (Scheme 5).

On treatment of **36** with potassium carbonate and benzyl bromide, the 4-benzyloxy methyl ester **46** was produced. For **49** this was then alkylated with isopropyl bromide and base to give the 3-isopropoxy-4-benzyloxy compound **47** (R^2^ = iPr) which was hydrogenated, alkylated with ethyl iodide and base and hydrolyzed to give rise to the benzoic acid **48** (R^2^ = iPr, R^3^ = Et). This benzoic acid **48** was then coupled to the aniline **38** (R^1^ = H) using T3P in ethyl acetate and triethylamine as a base, followed by hydrolysis with lithium hydroxide to provide the final compound **49**. The other non-identical di-alkoxy compounds **50**–**57** and **59** were similarly prepared via their corresponding benzoic acids **48 (**Scheme 5).

## 3 Results and discussion

**Fig. 2:**
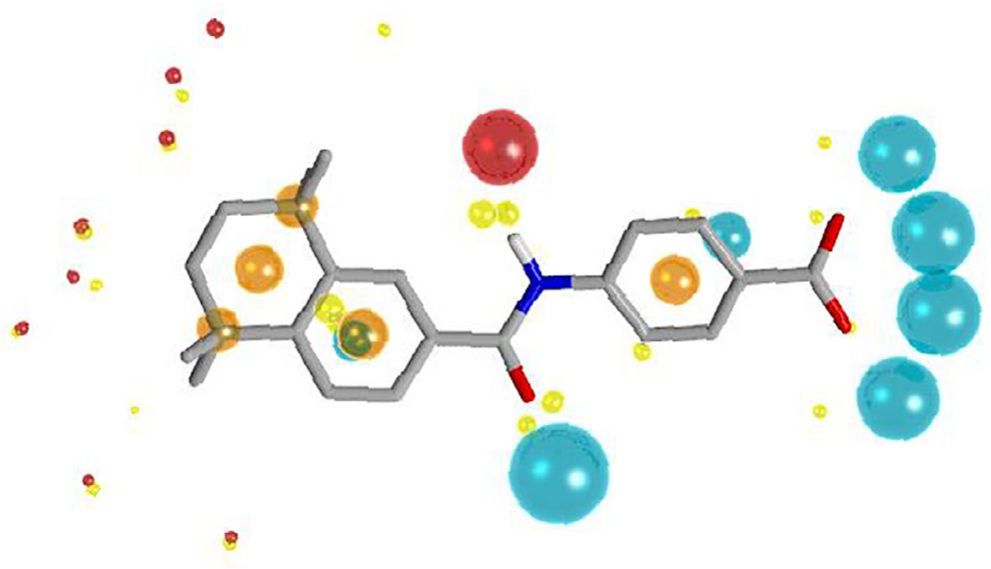
Cresset FieldScreen representation of bioactive conformation of AM580. (Blue field points (spheres) highlight energy minima for a positively charged probe, red for a negative probe. Yellow spheres represent an attractive van der Waals minima for a neutral probe and orange spheres represent hydrophobic centroids. Oxygen atoms are shown in red, nitrogen in blue. The size of the points is related to the strength of the interaction).

A ligand-based virtual screening approach, which ranks compounds by their similarity towards known active ligands, was adopted in a search for a novel chemical series of small molecule RAR*α* agonists. The extended electron density representation offered by the Cresset XED force-field provides a way to characterize the calculated field around a molecule.^14^ The subsequent molecular comparison uses four different 3D fields, positive and negative charge, steric shape and hydrophobicity, and allows a complete 3D conformational analysis of compounds to be performed.^15^, ^16^ The crystal structure of the selective RAR*α* antagonist BMS 195614 (**1)** in the human RAR*α* active site^17^ was overlaid with AM 580 (**2**), the antagonist removed and the resulting complete assembly minimized to give the putative bioactive conformation of AM 580 (**2)**. This procedure was also performed for AGN 193836 (**3**) to get its bioactive conformation. Molecular fields were added to each of these bioactive conformations (Fig. 2).

These unique molecular field patterns were used to search Cresset’s database of 2.5 M commercially available molecules, and the results ranked in similarity to the initial bioactive conformations (see Supplementary data for further details).

This methodology identified 3000 commercially available compounds as possible hit compounds. The 200 compounds that had the highest field overlays, Lipinski likeness, and synthetic tractability, were purchased. These were tested in transactivation assays at the RAR*α*, *β* and *γ* receptors. Full dose-response curves were generated for each active agonist, and the potency of each compound was expressed as a ratio of its EC_50_ compared to that of reference ATRA EC_50_ value generated on each 96 well plate. This produced several potent hits, including the lead compound **5** (Table 1). The 3,5-dichloro-4-ethoxy derivative **5** was considered to be one of the better starting points for a lead optimization exercise, not only because of its potency as an RAR*α* agonist but also because of its good selectivity over the RAR*β* and RAR*γ* receptors, with moderate lipophilicity (cLog P = 4.4) compared to AGN 195183 (**4**) (cLog P = 7.2). In addition, it had no systematic Cyp450 liability (inactive at 25 μM at Cyp1A, 2C19, 2C9, 2D6 and 3A4 isoforms), and was not cytotoxic in COS-7 cells (i.e. showed <20% cell death @ 50 × EC_50_ at the RAR alpha receptors).

**Table 1:** Potency and Selectivity of 3,5-dichloro-4-alkoxy RARα agonists.

**Figure:**
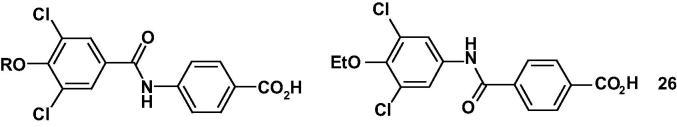

		Subtype-specific transactivation^a^					
		Relative EC_50_^b^					
Compd	RO	RAR*α*	RAR*β*	*β*/*α* ratio^c^	RAR*γ*	*γ*/*α* ratio^c^	cLogP^f^
**4**	**AGN195183**	**11**	1564	**141**	9836	**867**	7.2
**5**	EtO	**24**	1917	**79**	>3,00,000	**>12**,**500**	4.4
**12**	PrO	**15**	139	**9.5**	1196	**82**	4.9
**13**	BuO	**84**	717	**8.5**	1477	**18**	4.6
**14**	^i^PrO	**7**^d^	1417	**205**	823	**119**	4.7
**16**	*^t^*BuO	**7**	2927	**426**	6250	**909**	5.1
**15**		**10**	342	**33**	4703	**452**	5.3
**26**	–	**30**	355	**12**	>1,08,000	**>3600**	4.4
**60**	H	**92**^d^	642	**7**	5000	**55**	4.2
**61**	MeO	**30**	9525	**318**	5850	**195**	3.9

	ATRA	**1.0**(1.51 nM)^e^	**1.0**(0.52 nM) e		**1.0**(0.22 nM) e		

a Transactivation assays for the RAR alpha, beta and gamma receptors were performed using each of the mouse RAR ligand binding domains, Subtype-specific activity is expressed in terms of relative EC_50_ which is the concentration of retinoid required to produce 50% of the maximal observed response, normalised relative to that of ATRA.

b Mean EC_50_ for each compound divided by the mean EC_50_ of ATRA. Values were obtained from three separate experiments. Errors in these assays are approximately 20% of the mean values.

c The relative EC_50_ ratios of *α* to *β* and *α* to *γ*.

d Compound behaves as a partial agonist relative to the amplitude of the normalizing ATRA output.

e Mean of ATRA EC_50_ (nM).

f cLog P values were calculated in ChemDraw.

Our aim was to increase the RAR*α* potency and selectivity over RAR*β* while retaining the excellent selectivity over RAR*γ* shown by **5** and achieve oral bioavailability in the rat. The target profile was RAR*α* potency (RAR*α* EC_50_/ATRA EC_50_ < 10) with a selectivity of 2 orders of magnitude over RAR*β* and 3 orders of magnitude over RAR*γ* with an oral bioavailability of >35% in the rat.

Initial SAR showed that the three aromatic substituents in **5** seemed important for potency as the disubstituted, 3,5-dichloro derivative **60** was less potent at RAR*α* and also less selective than the 3,5-dichloro-4-ethoxy derivative **5** at RAR*β* and RAR*γ.* This helped focus our SAR on derivatives with a 3,4,5 substituted aromatic ring.

### 3.1 4-Substituted derivatives

We initially concentrated on the 4-substituent (Table 1). Increasing the length of the 4-alkoxy substituent to *n-*propoxy **12** and *n*-butoxy **13** resulted in a loss of selectivity at RAR*β* and RAR*γ*.

Increasing the bulk of the 4-alkoxy substituent to isopropoxy **14** and *tert*-butoxy **16** resulted in an increase in potency at RAR*α* and an increase in selectivity over RAR*β* but a loss of selectivity at RAR*γ*. In contrast, the cyclopentoxy compound, **15** was less selective than **5** at both RAR*β* and RAR*γ*.

We also explored the reverse amide **26** of **5** which lost significant selectivity against RAR*β* when compared to **5** and hence further work on the reverse amides was curtailed.

We next investigated the PK profile of these 3,5-dichloro-4-alkoxy derivatives. We used intrinsic clearance figures in mouse and human microsomes as a simple in vitro screen to minimize the risk of Phase 1 metabolism, before progressing to in vivo studies. The PK profile of the 3,5-dichloro-4-alkoxy series of compounds was poor. The ethoxy **5**, *tert*-butoxy **16** and cyclopentoxy **15** derivatives all had a high mouse, and moderate human intrinsic clearance and **15** was poorly orally absorbed with very low oral bioavailability in the rat (Table 2).

**Table 2:** In vitro and in vivo PK.

Compd	aLog D pH 7.4	LE^b^	intrinsic Cl_int_^c^		rat pK^d^		
			mouse (µL/min/mg protein)	human (µL/min/mg protein)	AUC po ng·min mL^−1^	Cl mL/kg/min	F%
**5**	1.7	0.45	127	18	ND	ND	ND
**15**	2.8	0.41	83	26	1674	2	0.3
**16**	2.6	0.45	91	16	ND	ND	ND
**18**	1.7	0.51	38	14	74,396	1.6	12
**31**	1.6	0.36	8	4	7,83,782	1	81
**39**	2.6	0.43	31	–	43,569	10	39
**49**	1.7	0.47	41	11	–	–	–
**51**	1.0	0.44	9	12	50,940	3	13

a Measured by octanol/buffer shake flask method at pH 7.4 (see Supplementary data file for details).^20^

b LE values were calculated by LE = (RT ln K_d_)/N, presuming EC_50_ ≈ K_d_.^18^

c Intrinsic clearance Cl_int_ data for screening purposes only: Mouse and Human microsomes were incubated with the test compound at 37 °C in the presence of the co-factor, NADPH. The data is the mean of 5 separate experiments. Compound disappearance monitored over 45 min period. SEM is less than 10% of the mean values.

d Rat PK (n = 4): AUC (ng·min mL^−1^) at 10 mg/kg, 8% Ethanol/92% PEG-400 formulation, Cl in mL min^−1^ kg^−1^. ND = not determined.

### 3.2 3,5-Disubstituted derivatives

To overcome these difficulties we turned our attention to the 3,5-sustituents in **5**. The patent analysis in this class of compounds showed that non-alkyl substituents in the 3,4,5-substituted aromatic ring of **5** appeared novel. With this in mind we analysed the medicinal chemistry parameters of the 3,5-substituents of our initial 4-OEt derivatives containing non-alkyl 3,5-substituents **5**, **62**, **63** and 3,5-dialkyl substituents **64** (Table 3). Ranking these four derivatives in terms of RAR*α* potency against the properties of the 3,5 substituents in the second aromatic ring, such as size (MR), lipophilicity (π) and electronic resonance (σ) (Table 3), shows that potency only increases with the lipophilicity π of the 3,5-sustituents (and not with the size or resonance effects of these substituents).

**Table 3:** 3,5-Disubstituted-4-ethoxy derivatives.

**Figure:**
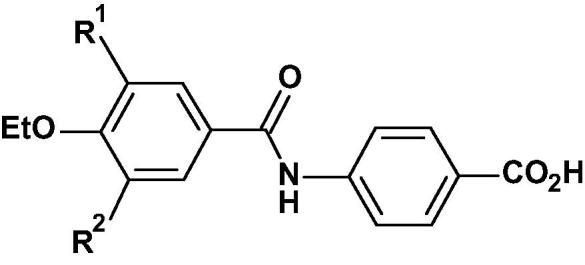

Compd	R^1^	R^2^	MR^a^ R^1^ + R^2^	π^b^ R^1^ + R^2^	σ^c^ R^1^ + R^2^	RAR*α rel* EC_50_^d^
**62**	EtO	EtO	25	0.76	0.2	370
**5**	Cl	Cl	12.06	1.42	0.74	24
**63**	Br	Br	17.76	1.72	0.78	5
**64**	*^t^*Bu	*^t^*Bu	39.24	3.96	-0.20	0.2
**31**	^i^PrO	^i^PrO	34.12	1.70	0.20	26^e^

a Sum of size (MR) of *meta* substituents R^1^ and R^2^.

b Sum of lipophilicity (π) of substituents R^1^ and R^2^.

c Sum of electronic resonance effect (σ) of *meta* substituents R^1^ and R^2^. For parameters see ref 19.

d Relative EC_50_ see^a,b^Table 1.

e Partial agonist see^d^Table 1.

A search of possible aromatic substituents showed that the isopropoxy group has a similar lipophilicity to a chlorine**/**bromine atom found in **5/63** and a similar size to a *tert*-butyl found in the more potent derivative **64**. This suggested that the 3,5-diisopropoxy derivative **31** should be at least as active as the chloro and bromo derivatives **5** and **63**, and why the 3,5-diethoxy analog **62** which is the least lipophilic, is the least active.

### 3.3 3,4,5-Trialkoxy and 3,4,-dialkoxy derivatives

Encouragingly **31** proved to have good RAR*α* potency (Table 3). In addition, **31** has high selectivity over RAR*β* and RAR*γ* (Table 4), and low mouse and human intrinsic clearance with excellent oral absorption and bioavailability (81%) in the rat (Table 2), although it was shown to be only a partial RAR*α* agonist. The close profile of **5** and **31** in terms of RAR*α* potency, as well as RAR*β* and RAR*γ* selectivity, shows that in this case, the iPrO group is a good bioisostere of the Cl group. This led the project away from the 3,5-dichloro template and enabled exploration of the alkoxy derivatives at these positions which give a lipophilic surface without the high lipophilicity of the similar sized tertiary butyl group seen in **64**, making the template more drug-like. Further analogs of this trialkoxy template **31** were investigated in an attempt to increase its alpha potency while maintaining the excellent beta and gamma selectivity as well as its good PK profile. Increasing the size of the 3,5-substituents in **31** to give the di-cyclopentoxy derivative **32** or increasing the size of the 4-substituents to give **33** maintained the good RAR*α* potency and RAR*β* selectivity but lost selectivity against RAR*γ* (Table 4). Decreasing the size of both the 3- and 5-isopropoxy groups to give the 3,4,5-triethoxy derivative **62**, resulted in a substantial loss of RAR*α* potency (Table 3). In addition **31**, **32** and **33** all exhibited some partial agonist activity at RAR*α*. However a close analog the 3,4-diethoxy-5-isopropoxy derivative **34** showed that it was possible to have full RAR*α* agonist properties with trialkoxy derivatives (Table 4).

**Table 4:** Potency and selectivity of 3,4,5-trialkoxy and 3,4,-dialkoxy RARα agonists.

**Figure:**
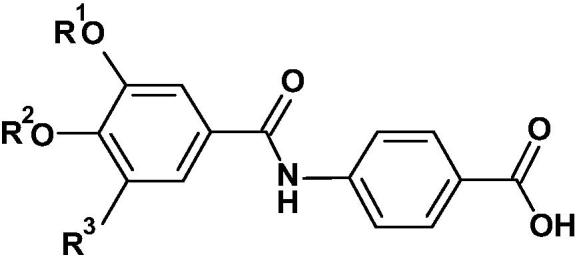

				Subtype-specific transactivation^a^				
				Relative EC_50_^b^				
Compd	R^1^O	R^2^O	R^3^	**RAR*α***	RAR*β*	***β*/*α*** **ratio**^c^	RAR*γ*	***γ*/*α*** **ratio**^c^
**62**	EtO	EtO	EtO	**368**	64,148	**174**	5882	**16**
**31**	^i^PrO	EtO	^i^PrO	**26**^d^	4560	**175**	56,900	**2190**
**32**		EtO		**29**^d^	4200	**145**	550	**19**
**33**	^i^PrO	^i^PrO	^i^PrO	**27**^d^	2600	**96**	225	**8**
**34**	^i^PrO	EtO	EtO	**29**	2450	**84**	960	**34**
**49**	^i^PrO	EtO	Cl	**0.7**^d^	103	**150**	8083	**11,721**
**50**		EtO	Cl	**1.0**^d^	115	**115**	1706	**1706**
**39**	^i^PrO	^i^PrO	Cl	**1.7**	89	**54**	1386	**838**
**40**			Cl	**2.4**^d^	53	**22**	1059	**447**
**41**	*^t^*BuO	*^t^*BuO	Cl	**0.9**	38	**44**	162	**189**
**42**			Cl	**1.7**^d^	55	**32**	571	**336**
**51**	^i^PrO	MeO	Cl	**5.3**	1500	**283**	10,833	**2043**
**52**	EtO	^i^PrO	Cl	**2.1**	7.1	**3.1**	1202	**570**

		ATRA		**1.0**(1.51 nM)^e^	**1.0**(0.52 nM)^e^		**1.0**(0.22 nM)^e^	

a Transactivation assays were performed using the RAR alpha, beta and gamma receptors containing each of the mouse RAR ligand binding domains. Subtype-specific activity is expressed in terms of relative EC_50_ which is the concentration of retinoid required to produce 50% of the maximal observed response, normalised relative to that of ATRA.

b The relative EC_50_ is the mean EC_50_ for each compound divided by the mean EC_50_ of ATRA. Values were obtained from three separate experiments. Errors in these assays are approximately 20% of the mean values.

c The relative EC_50_ ratios of *α* to *β* and *α* to *γ*.

d Compound behaves as a partial agonist relative to the amplitude of the normalizing ATRA output.

e Mean of ATRA EC_50_ (nM).

This unsymmetrical derivative was further exploited by the investigation of a series of 3,4-alkoxy derivatives (Table 4). Replacing one of the isopropoxy groups in the lead **31** with a chloro atom gave the chloro-dialkoxy derivative **49** which had increased potency at RAR*α* and also maintained the excellent selectivity at RAR*β* and RAR*γ.* However, this compound was also only a partial agonist at RAR*α*.

Increasing the size of the 3-isopropoxy in **49**to 3-cyclobutyl in **50** gave no change in profile. However, increasing the 4-ethoxy group in **49** to the 4-isopropoxy in **39** gave a similar level of potency at RAR*α* as a full agonist. The molecule was also an order of magnitude more potent than **31** at RAR*α* while maintaining excellent selectivity at RAR*γ* with moderate selectivity at RAR*β*. In addition, the di-isopropoxy derivative **39** was orally well absorbed in the rat with a bioavailability of 39% (Table 2). Thus **39** satisfied our target profile except for selectivity at RAR*β*. Increasing the size of the alkoxy groups to the di-cyclobutyl in **40**, di-*tert-*butyl in **41** and di-cyclopentyl in **42** maintained potency at RAR*α*, but decreased selectivity at RAR*β* and RAR*γ*. Interestingly reducing the size of the 4-ethoxy in **49** to 4-methoxy in **51** gave a full agonist with good RAR*α* potency and selectivity at RAR*β* and RAR*γ*. However, it had a low oral bioavailability (13%) in the rat (Table 2).

### 3.4 Substitution of the benzoic acid ring

*Ortho*-fluoro substitution in the benzoic acid ring of the bicyclic 5,5,8,8-tetramethyl-5,6,7,8-tetrahydronaphthalene analogs which lead to AGN 195183 (**4**)^10^has been shown to increase RAR*α* binding potency and increase selectivity over RAR*β* and RAR*γ* in the transactivation assay.

Based on this precedent, a series of *ortho*-substituted benzoic acid derivatives of our initial lead template **5** were prepared (Table 5). While the *ortho*-fluoro substitution product **17** maintained potency and selectivity, the *ortho*-methyl substitution product, **18** improved RAR*α* potency 20-fold and maintained good RAR*β* and RAR*γ* selectivity. In addition, **18** had a lower mouse and human intrinsic clearance, as well as a somewhat improved bioavailability (12%) in the rat (Table 2), compared to the unsubstituted benzoic acid derivative **5**. Compounds **19**, **20** and **21** with larger substituent groups, either lost RAR*α* potency or RAR*β***/**RAR*γ* selectivity compared to **5**. As a result of these findings, a series *ortho*-methyl and *ortho*-fluoro substituted benzoic acid derivatives of the 3,4-dialkoxy-5-chloro template were prepared (Table 6). The initial trend from the **5** series (Table 5) was also seen in the 3,4-dialkoxy-5-chloro series (Table 6).

**Table 5:** *Ortho*-substituted benzoic acid derivatives of **5**.

**Figure:**
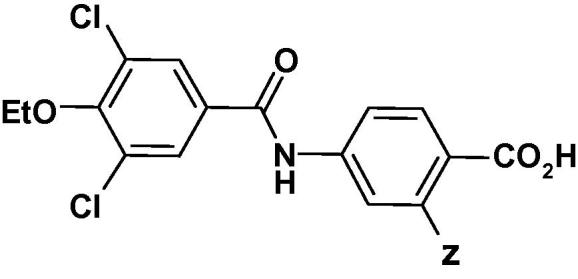

Compd	Z	RAR*α* *rel* EC_50_^a^	*β*/*α* ratio^b^	*γ*/*α* ratio^b^
**5**	H	24	80	>12,500
**17**	F	25	80	>80
**18**	CH_3_	0.9	82	151
**19**	OH	33^c^	37	149
**20**	Cl	151	64	>50
**21**	CF_3_	1.7^c^	1.5	11

^a,b^ and ^c^ see Table 1.

**Table 6:** *Ortho-*fluoro and *ortho*-methyl (3-chloro-4,5-dialkoxybenzamido)benzoic acids.

**Figure:**
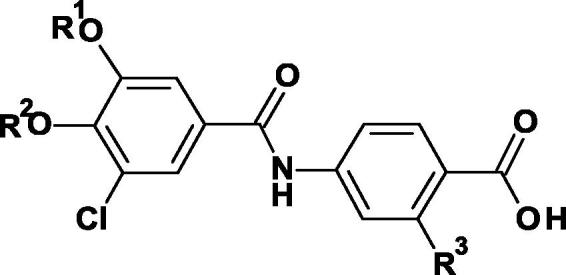

				Potency and Selectivity				PK				
Compd	R^1^O	R^2^O	R^3^	RAR*α* *rel* EC_50_^a^	*β*/*α* ratio b	*γ*/*α* ratio^b^	LogD_pH 7.4_^d^	intrinsic Cl_int_^e^		rat po^f^		
								mouse	human	AUC	Cl	F%
**53**	^i^PrO	MeO	F	6.7	609	17,600	1.0	11	0.3	10,871	15	15
**54**	^i^PrO	EtO	F	1.1	487	6169	1.4	17.9	3.3	15,376	12	12
**43**	^i^PrO	^i^PrO	F	2.7	34	300	2.0	18.8	6.9	–	–	–
**55**	^i^PrO	MeO	Me	4.7^c^	241	509	1.3	10.6	1.5	–	–	–
**44**	^i^PrO	^i^PrO	Me	0.97	45	2947	2.3	26.2	15	77,670	5.4	66
**56**	^i^PrO	EtO	Me	1.6	200	11,000	1.8	37.6	5.3	70,765	7	40
**57**	EtO	^i^PrO	Me	7	286	1675	1.9	25.7	8.7	–	–	–
**58**	MeO	EtO	Me	33	210	>250	–	18.4	–	–	–	–
**59**		EtO	Me	2.6	58	38,461	–	42.3	–	–	–	–
**45**	*^t^*BuO	*^t^*BuO	Me	0.64	13,200	128	2.9	36.2	28.7	–	–	–

a Relative EC_50_.

b Relative EC_50_ ratios and ^c^partial agonist see Table 1.

d Measured by octanol/buffer shake flask method at pH 7.4 see Table 2.

e Cl_int_ (µL/min/mg protein) and ^f^AUC (ng·min mL^−1^), Cl (mL min^−1^ kg^−1^) see Table 2.

The *ortho*-fluoro substituted derivatives **53**, **54** and **43** (Table 6) maintained RAR*α* potency and selectivity compared to their corresponding unsubstituted derivatives **51**, **49** and **39** and, in addition, had a lower mouse and human intrinsic clearance with the latter being in single figures. However, although both **53** and **54** met our target profile in terms of high RAR*α* potency with a selectivity of 2 orders of magnitude over RAR*β* and 3 orders of magnitude over RAR*γ*, they both had low oral bioavailability (15% and 12% respectively) in the rat. The *ortho*-methyl substituted derivative **55** had a similar mouse and lower human intrinsic clearance (Table 6) compared to the unsubstituted derivative **51** (Table 2). However, it had only partial RAR*α* agonist activity. Both the *ortho*-methyl derivatives **56** and **44** had good bioavailability (≥40%) in the rat and lower mouse intrinsic clearance (Table 6) compared to the unsubstituted derivatives **49** and **39** (Table 2), with **56** having the lowest (single figure) human intrinsic clearance of these four derivatives.

While both derivatives **56** and **44** had high RAR*α* potency and good bioavailability **56** was superior in terms of selectivity at RAR*β* (2 orders of magnitude) and RAR*γ* (4 orders of magnitude) and possessed a better overall potency, selectivity and PK profile than the other analogs **45**, **57**–**59** shown in Table 6.

The 3-OEt, 4-OiPr geometrical isomer **57** was less potent and less selective at RAR*γ* than **56** which is analogous to the trend seen with compounds **51** and **49** in the unsubstituted benzoic acid series. This emphasizes the need for a more lipophilic group than OEt in the 3-and 5-position in this template which was initially seen in Table 3. Thus the 3-OiPr, 4-OEt derivative **56** reached our target profile in terms of potency, selectivity, and oral bioavailability.

The excellent RAR*α* potency, good RAR*β* and RAR*γ* selectivity and PK profile of the full agonist **56** suggested further investigations to see if it had sufficient drug-like properties to be an orally bioavailable, highly potent and selective RAR*α* agonist with therapeutic potential.

### 3.5 Predevelopment studies of 4-(3-chloro-4-ethoxy-5-isopropoxybenzamido)-2-methylbenzoic acid **56**

#### 3.5.1 ADME profiling

Predevelopment ADME studies revealed that **56** has a good Cyp 450 profile with no significant inhibition IC_50_ > 25 μM against five Cyp 450 isozymes (1A2, 2C9, 2C19, 2D6, 3A4), and has a human and mouse plasma protein binding of 93% and 91% respectively.^20^

Compound **56** has also been examined by CEREP in a panel of 120 other receptors, channels and enzymes. The compound at 10 μM demonstrated no significant interactions with any of the sites examined leaving a window of some 4 orders of magnitude between its actions at RAR and any non-RAR site.^21^ The highest inhibition of 25% was found for the 5HT2B site. To exclude potential cardiovascular side effects, compound **56** was tested in vitro on the cardiac hERG channel and did not show any significant binding to hERG up to the concentration of 10 μM.^20^

#### 3.5.2 Hepatocyte stability

We initially used a microsomes assay as a screen to rank order compounds of interest in terms of their metabolic stability. As microsomes only contain phase I metabolising enzymes it was of interest to screen our lead compound **56** in a secondary screen using hepatocytes which contain the full complement of drug metabolising enzymes present in the liver.

The metabolic stability of compound **56** was tested at two concentrations (1 μM and 30 μM) in mouse, rat, dog, Cynomolgus monkey and human cryopreserved hepatocytes. The compound was shown to be stable, with a long t½ and low clearance in all species (Table 7), which correlates with the available PK data (Table 8).

**Table 7:** Stability of **56** in hepatocytes.

Conc (μM)	Species	Half-life (minutes) a	Cl_int_ (μl/min/million cells)
1	Mouse	224	12
	Rat	357	4
	Dog	>450	<3
	Cynomolgus monkey	>450	<3
	Human	>450	<3
30	Mouse	>300	<9
	Rat	>450	<3
	Dog	>450	<3
	Cynomolgus monkey	>450	<3
	Human	>450	<3

a Data are expressed as mean values (n = 2). For assay details see Supplementary data file.

**Table 8:** Pharmacokinetic Profiles of **56** in Mice and Dogs.^a^

	iv			po		
Species	Cl (mL/h/kg)	Vss (mL/kg)	t_1/2_ (h)	C_max_ (ng/mL)	T_max_ (h)	F (%)
mice^b^	4.7	0.3	1.9	2007	0.25	84
dog^c^	2.3	0.66	9.2	2050	0.5	83

a Administered at a dose of 1 mg/kg by both iv and po routes in mice. Administered at a dose of 0.5 mg/kg, iv, 1 mg/kg, po, in dogs. Vehicle = 2% DMSO in 0.05 M phosphate buffered saline, pH 7.4. Data are expressed as mean values (mice, n = 3. dogs n = 3).

b C57 mice. For assay details see Supplementary data file.

c Beagle dogs. For assay details see Supplementary data file.

#### 3.5.3 PK profile in mice and dogs

The PK profile of **56** was also studied in mice and dogs (Table 8). Compound **56** showed low plasma clearance (Cl) and low volume of distribution (Vss), resulting in sustained plasma half-lives in each species (iv t_1/2_: mice, 1.9 h; dog, 9.2 h). In addition, oral administration of **56** exhibited high bioavailabilities >80% in both mice and dogs. These results encouraged us to investigate **56** further as a predevelopment candidate.

#### 3.5.4 Human RAR alpha receptor

As we planned to perform PK and further in vivo evaluation in rodents, we initially used the corresponding in vitro transcriptional transactivation assays with gal4 fusion receptor constructs, created using each of the mouse RAR ligand-binding domains. Although the percentage identity of amino acid sequences between the mouse and human RAR ligand-binding domains of all three RAR types (α,β or γ) is 99–100%,^23^ we thought it prudent to confirm the activity and selectivity of our lead compound **56** against the human RAR ligand-binding domains in a transcriptional transactivation assay before further predevelopment studies were investigated. We also tested an earlier less active analog **15** from the 3,5-dichloro template, and AM 580 (**2)** for comparison (Table 9).

**Table 9:** Human and Mouse RARα Potency plus β and γ selectivity.

**Figure:**
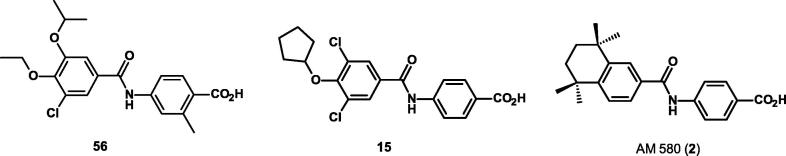

Property	56		15		AM580(2)		ATRA	
	mouse^a^	human^b^	mouse^a^	human^b^	mouse^a^	human^b^	mouse	human
RAR*α rel* EC_50_	1.6	0.59	10.4	8.1	0.02	0.13	1.0 (1.51 nM)^c^	1.0 (1.01 nM)^d^
Selectivity *β*/*α* ratio	200-fold	290-fold	33-fold	289-fold	1130-fold	162-fold	0.34-fold	0.33-fold
Selectivity *γ*/*α* ratio	11,000-fold	>13,000-fold	452-fold	2322-fold	826-fold	505-fold	0.15-fold	0.11-fold

a See Table 1.

b Transactivation RAR human assay. For details see Supplementary data file.

c Mean of ATRA EC_50_ (nM) mouse assay RAR*α.*

d Mean of ATRA EC_50_ (nM) human assay RAR*α.*

There is a good correspondence for RAR*α* potency between human vs mouse for **56** and **15** with the human being slightly more potent, in contrast to the RAR*α* potency for AM 580 (**2)** where the human is less potent than the mouse (Table 9). Similarly, the *α* vs *β* selectivity comparison for **56** and **15**, shows that the human is more selective than the mouse, while for AM 580 (**2)** the human is less selective than the mouse. Also, *α* vs *γ* selectivity for **56** is 4 orders of magnitude compared to AM 580 (**2)** where it is only 2 orders of magnitude for both human and mouse.

#### 3.5.5 In vitro toxicology

In common with most of the other compounds in the series, the lead compound **56** showed no cytotoxicity in COS-7 cells at a 50-fold multiple of its EC_50_.^20^ When examined in a high content cell toxicity screen in HEPG2 cells (Cyprotex), **56** was found to have no effect at concentrations up to 50 μM on cell or mitochondrial viability markers.^20^ This is in contrast to the more lipophilic molecule AM 580 (**2)** which caused a significant increase in cell membrane permeability and a significant decrease in mitochondrial membrane potential at concentrations between 10 and 30 μM.

When **56** was examined for genetic toxicity, it was negative in bacterial cytotoxicity tests up to 100 μM, negative in an Ames test in three bacterial strains and in an in vitro micronucleus test in CHO-K1 cells, in all cases in both the presence and absence of S9.^21^ In the absence of S9 it should be noted that AM 580 (**2)**, the reference RAR*α* agonist has been shown by others to be a mutagen in vitro.^11^, ^12^

#### 3.5.6 Ease of synthesis

The 4-(3-chloro-4-ethoxy-5-isopropoxybenzamido)-2-methylbenzoic acid **56** can be synthesized in 9 high yielding reaction steps from 3-chloro-4-hydroxy-5-methoxybenzoic acid (**35**) (Scheme 5). It is available as a stable highly crystalline, non-hygroscopic, white powder with a melting point of 186 °C, and with a solubility of >5 mg/mL, as the sodium salt in water at 35 °C.

#### 3.5.7 Profile of lead compound **56**

The 3-OiPr, 4-OEt, 5-Cl *ortho* methyl benzoic acid derivative **56** met our target profile in terms of high RAR*α* agonist potency with a high degree of selectivity over RAR*β* (of 2 orders of magnitude) and excellent selectivity over RAR*γ* (4 orders of magnitude) at both the mouse and human receptors. It has high levels of potency in the RAR*α* binding assay (IC_50_) showing that the transactivation activity observed was being mediated through the alpha receptor (Table 10). As expected **56** was also selective vs RXR (IC_50_ > 10 μM in human RXR α and β binding assays).^22^ It also possesses good drug-like properties, a low human intrinsic clearance (5.3 µL/min/mg protein) in microsomes and a measured Log D = 1.8, which resulted in good oral exposure with low clearance and good bioavailability (40%) in the rat (Table 10). In contrast, both **15** and **2** have human intrinsic clearance in double figures and a higher Log D = 2.8, which resulted in low oral exposure in the rat with low bioavailability (0.3%) for **15**. Compound **56** was also shown to be metabolically stable to hepatocytes with a long t½ and low clearance in human and 4 animal species (Table 7) together with a high bioavailability (>80%) in both mice and dogs with low plasma clearance (CL) and a sustained plasma half-live (iv t_1/2_: mice, 1.9 h; dog, 9.2 h) (Table 8). In addition **56** has a solubility of >5 mg/mL as the sodium salt, no systematic Cyp 450 liability against five isoforms (1A2, 2C9, 2C19, 2D6, 3A4) and demonstrated no inhibition (at 10 μM) in a binding assay for hERG channels. It was not cytotoxic in COS-7 cells and was negative for genetic toxicity in the Ames test and micronucleus test in CHO-K1 cells.

**Table 10:** Comparison of the RAR*α* Agonist Potency, selectivity versus the RAR*β* and RAR*γ* Human and Mouse Receptors, Human Intrinsic Clearance and Pharmacokinetic Profile in Rat for **56** and **15**.

Binding Activity		Agonist Potency and Selectivity			PK				
Compd	RAR*α* *rel* IC_50_^a^	RAR*α* *rel* EC_50_ bm/chu	*β*/*α* ratio bm/chu	*γ*/*α* ratio bm/chu	intrinsic Cl_int_^d^	rat po^e^			f Log D pH 7.4
					human	AUC	Cl	*F*%	
**56**	3.6	1.6/0.6	200/298	11,000/>13,000	5.3	70,765	7	40	1.8
**15**	115	10.4/8.1	33/289	452/2322	26	1674	2	0.3	2.8
AM 580(**2)**	9	0.02/0.13	1130/162	826/505	15.6	–	–	–	2.8

a RAR*α* binding assay. The relative IC_50_ is the mean IC_50_ for each compound divided by the mean IC_50_ of ATRA (IC_50_ = 0.6 nM). Values were obtained from three separate experiments.

b m = mouse receptor, see Table 1.

c hu = human receptor, see Table 9.

d Human microsomes Cl_int_ (µL/min/mg protein).

e AUC po ng·min mL^−1^,Cl mL/kg/min.

f Log D see Table 2.

## 4 Conclusions

We have used a ligand-based virtual screening exercise based on the bioactive conformation of AM 580 (**2)** and AGN 193836 (**3)** to identify the novel, less lipophilic RAR*α* agonist 4-(3,5-dichloro-4-ethoxybenzamido) benzoic acid **5**, which has good selectivity over the RAR*β*, and RAR*γ* receptors. Analysis of the medicinal chemistry parameters of the 3,5-substituents of derivatives of template **5** showed that RAR*α* potency is driven by the lipophilicity of these substituents. It showed that the iPrO group is a good bioisostere of the Cl group in this case and that the 4′-(3,5-diisopropoxy-4-ethoxybenzamido)benzoic acid derivative **31** has a close profile to **5** in terms of RAR*α* potency as well as RAR*β* and RAR*γ* selectivity. The low mouse and human intrinsic clearance with excellent oral absorption and bioavailability (81%) in the rat shown by **31** led to the exploration of the more drug-like branched dialkoxy derivatives, the best of which was the 4-(3-chloro-4,5-diisopropoxybenzamido)benzoic acid derivative **39** which was an order of magnitude more potent than **31** at RAR*α*, while maintaining excellent selectivity over RAR*γ* with moderate selectivity at RAR*β* and was orally well absorbed in the rat with a bioavailability of 39%. Substitution at the *ortho*-position of benzoic acid **5**, with a range of groups, has shown that methyl groups are the best at increasing potency while maintaining good RAR*β* and RAR*γ* selectivity. Methyl substitution at the *ortho*-position of the 4′-benzoic acid ring of a series of 4′-(3-chloro-4,5-dialkoxybenzamido)benzoic acid derivatives gave the novel RARα agonist 4-(3-chloro-4-ethoxy-5-isopropoxybenzamido)-2-methylbenzoic acid **56** as the best in terms of RAR*α* agonist potency and selectivity versus RAR*β* (2 orders of magnitude) and RAR*γ* (4 orders of magnitude) at both the human and mouse RAR receptors. This potent RAR*α*-specific agonist with improved physicochemical properties also has high bioavailability (>80%) in both mice and dogs with a good PK profile and drug-like properties and was shown to be negative in the cytotoxicity and genotoxicity screens warranting further consideration as a potential therapeutic agent.

## 5 Experimental procedures

All starting materials and solvents, as well as compounds **5**, **60**, **61** and **62**, were obtained from commercial sources. Hydrogenations were performed either on a Thales H-cube flow reactor or with a suspension of the catalyst under a balloon of hydrogen. Microwave reactions were carried out on a Personal Chemistry Smith Synthesizer Workstation with a 300 W single mode microwave cavity. Ion exchange chromatography was performed using strong cation exchange resin (SCX) cartridges purchased from Sigma-Aldrich and washed with methanol prior to use. The reaction mixture to be purified was first dissolved in methanol and then loaded directly onto the SCX and washed with methanol. The desired material was then eluted by washing with 1% NH_3_ in methanol. Silica gel column chromatography was performed using Silicycle pre-packed silica (230–400 mesh, 40–63 μM) cartridges. Preparative HPLC was carried out using a Gilson HPLC and an Agilent 5 µm Prep-C18 21.2 × 50 mm column. Detection was achieved using a UV detector at 254 nm. Mobile phase A: 0.1% aqueous formic acid, Mobile phase B: 0.1% formic acid in methanol. A flow rate of 40 mL/min was used and a gradient employed as follows; 0.0–0.8 min 5% B; 0.8–7.3 min 5–95% B; 7.3–8.3 min 95% B; 8.3–8.4 min 95–5% B. Analytical LCMS was performed using an Agilent 1200 HPLC and mass spectrometer system with a Scalar 5 µm C18 4.6 × 50 mm column and peaks detected by positive or negative ion electrospray ionization and a UV detector at 254 nm. All tested compounds were found to be of ≥95% purity using analytical LCMS. ^1^H and ^13^C NMR spectra were recorded using a Bruker Avance III ^TM^ 400 spectrometer at 400 and 110 MHz respectively, using either residual non-deuterated solvent or tetramethylsilane as a reference in the various solvents specified. All animal studies were ethically reviewed and carried out in accordance with the United Kingdom Animals (Scientific Procedures) Act 1986 by CXR Biosciences Ltd, James Lindsay Place, Dundee Technopole, Dundee DD 5JJ.

### 5.1 Chemistry

#### 5.1.1 4-(3,5-Dichloro-4-(cyclopentyloxy)benzamido)benzoic acid (**15**)

*Step (i)*: **Methyl 3,5-dichloro-4-(cyclopentyloxy)benzoate** Methyl 3,5-dichloro-4-hydroxybenzoate **6** (1.00 g, 4.52 mmol) was dissolved in *N, N*-dimethylformamide (8 mL) and treated with bromocyclopentane (534 µL, 4.98 mmol), followed by potassium carbonate (937 mg, 6.79 mmol). The mixture was stirred at 80 °C for 3 h and then partitioned between ethyl acetate (100 mL) and water (100 mL). The aqueous phase was extracted with ethyl acetate (50 mL) and the combined organic phases washed successively with water (5 × 50 mL) and brine (50 mL), then dried over magnesium sulfate and filtered. The solvent was removed in vacuo to afford methyl 3,5-dichloro-4-(cyclopentyloxy) benzoate (1.10 g, 84% yield). ^1^H NMR (400 MHz, CDCl_3_) *δ* 7.97 (2H, s), 5.04 (1H, m), 3.90 (3H, s), 2.04–1.91 (4H, m), 1.82–1.75 (2H, m), 1.69–1.60 (2H, m).

*Step (ii)*: **3,5-Dichloro-4-(cyclopentyloxy)benzoicacid (7: R^1^** = **cyclopentyloxy)**. Methyl 3,5-dichloro-4-(cyclopentyloxy)benzoate (1.05 g, 3.63 mmol) and lithium hydroxide (174 mg, 7.26 mmol) were combined in tetrahydrofuran (10 mL), and water (1.5 mL) was added dropwise until a solution formed. The resultant mixture was stirred at room temperature for 12 h. The tetrahydrofuran was removed in vacuo and the residue acidified using aqueous 1 M hydrochloric acid. The resultant precipitate was filtered to afford 3,5-dichloro-4-(cyclopentyloxy)benzoic acid (**7**: R^1^ = cyclopentyloxy), (820 mg, 82% yield). ^1^H NMR (400 MHz, DMSO‑*d_6_*) *δ* 8.10 (2H, s), 5.03 (1H, m), 2.04–1.91 (4H, m), 1.82–1.75 (2H, m), 1.69–1.60 (2H, m).

*Step (iii)*: **Methyl 4-[3,5-dichloro-4-(cyclopentyloxy)benzamido]benzoate (10: R^1^** = **cyclopentyloxy, R^2^ = H, R = Me)**. A solution of **(7:** R^1^ = cyclopentyloxy**)** (100 mg, 363 µmol) in dichloromethane (5 mL), cooled to 0 °C, was treated with oxalyl chloride (63.6 µL, 727 µmol), followed by a drop of *N, N*-dimethylformamide. The resultant mixture was stirred for 1 h at room temperature. The solvent was evaporated in vacuo and the residue dissolved in dichloromethane (5 mL) and then treated with a solution of methyl 4-aminobenzoate (**8**: R^2^ = H, R = Me) (54.9 mg, 363 µmol) and di-isopropylethylamine (190 µL, 1.09 mmol) in dichloromethane (5 mL). The reaction mixture was stirred for12 h at room temperature and then partitioned between dichloromethane (20 mL) and aqueous 1 M hydrochloric acid (20 mL). The phases were separated, and the organic phase was washed successively with water (2 × 20 mL), and brine (20 mL), dried over magnesium sulfate, filtered and then the solvent was removed in vacuo. The residue was purified by silica gel chromatography (12 g, 0–100% ethyl acetate/isohexane) to afford methyl 4-[3,5-dichloro-4-(cyclopentyloxy)benzamido]benzoate (**10**: R^1^ = cyclopentyloxy, R^2^ = H, R = Me), **(**30 mg, 20% yield). 1H NMR (400 MHz, CDCl_3_) *δ* 8.06 (2H, d, *J* = 8.8 Hz,), 7.85 (1H, br s), 7.82 (2H, s), 7.71 (2H, d, *J* = 8.8 Hz), 5.07–5.03 (1H, m), 3.92 (3H, s), 2.10–1.90 (4H, m), 1.85–1.70 (2H, m), 1.70–1.60 (2H, m).

*Step (iv)*: **4-[3,5-Dichloro-4-(cyclopentyloxy)benzamido]benzoicacid (15)**. Compound **(10:** R^1^ = cyclopentyloxy, R^2^ = H, R = Me**),** (30.0 mg, 73 µmol) and lithium hydroxide (3.5 mg, 0.147 mmol) were combined in tetrahydrofuran (3 mL) and water was added dropwise until a solution formed. The resultant mixture was stirred at room temperature for 16 h. The tetrahydrofuran was removed in vacuo and the residue acidified using aqueous 1 M hydrochloric acid. The resultant precipitate was filtered to afford 4-[3,5-dichloro-4-(cyclopentyloxy)benzamido]benzoic acid **15** (15.0 mg, 51% yield) as a white solid. ^1^H NMR (400 MHz, DMSO‑*d*_6_) *δ* 12.77 (1H, s), 10.58 (1H, s), 8.07 (2H, s), 7.93 (2H, d, *J* = 8.8 Hz,), 7.88 (2H, d, *J* = 8.8 Hz), 5.06–5.01 (1H, m), 1.90–1.60 (8H, m). *m*/*z* 392 (M−H)^−^ (ES^−^).

The compounds **12**–**14**, **17, 20, 21, 63** and **64** were similarly prepared as **15**: see Supplementary data for experimental and spectroscopic details.

#### 5.1.2 4-(4-(*tert*-Butoxy)-3,5-dichlorobenzamido)benzoic acid (16)

*Step (i)*: **Methyl 4-(benzyloxy)-3,5-dichlorobenzoate**. Crude methyl 4-(benzyloxy)-3,5-dichlorobenzoate (16.9 g) was prepared from methyl 3,5-dichloro-4-hydroxybenzoate (**6**) (10 g, 45.2 mmol) and benzyl bromide (15.5 g, 90 mmol) using a procedure essentially the same as in *step (i)* for **15**, except that the mixture was stirred at room temperature for 18 h. The crude product was partially purified by silica gel chromatography (330 g, 0–10% EtOAc/isohexane) to afford a white solid. The material was used in the next step without further purification.

*Step (ii)*: **4-(Benzyloxy)-3,5-dichlorobenzoicacid (7: R^1^** = **CH_2_Ph)**. 4-(Benzyloxy)-3,5-dichlorobenzoic acid (**7**: R^1^ = CH_2_Ph) (12.8 g, 96% over 2 steps) was prepared from crude 4-(benzyloxy)-3,5-dichlorobenzoate (16.9 g) using a procedure essentially the same as in *step (iv)* for **15**: ^1^H NMR (400 MHz, DMSO‑*d*_6_) *δ* 7.88 (2H, s), 7.56–7.48 (2H, m), 7.44–7.37 (3H, m), 5.05 (2H, s). *m*/*z* 295 (M−H)^−^ (ES^−^).

*Step (iii)*: **Methyl 4-(4-(benzyloxy)-3,5-dichlorobenzamido)benzoate (10: R^1^** = **CH_2_Ph, R^2^ = H, R = Me)**. Methyl 4-(4-(benzyloxy)-3,5-dichlorobenzamido)benzoate (**10**: R^1^ = CH_2_Ph, R^2^ = H, R = Me) (9.81 g, 51%) was prepared from 4-(benzyloxy)-3,5-dichlorobenzoic acid (**7**: R^1^ = CH_2_Ph) (12.8 g, 43.2 mmol) using a procedure essentially the same as in *step (iii)* for **15**, except the crude product was crystallized from isohexane/EtOAc to afford the product as a white solid. ^1^H NMR (400 MHz, CDCl_3_) *δ* 8.07 (2H, d, *J* = 8.8 Hz), 7.84 (2H, s), 7.73 (2H, d, *J* = 8.8 Hz), 7.59–7.52 (2H, m), 7.44–7.36 (3H, m), 5.13 (2H, s), 3.92 (3H, s). *m*/*z* 428 (M−H)^−^ (ES^−^).

*Step (iv)*: **Methyl 4-(3,5-dichloro-4-hydroxybenzamido)benzoate(11: R^2^=H)**. A solution of methyl 4-(4-(benzyloxy)-3,5-dichlorobenzamido)benzoate (**10**: R^1^ = CH_2_Ph, R^2^ = H, R = Me) (8.8 g, 20.5 mmol) in DCM (500 mL) was cooled to 0 °C and treated dropwise with boron trichloride (20.5 mL, 20.5 mmol, 1 M in DCM). The mixture was then allowed to stir at room temperature for 12 h. The mixture was cooled in an ice bath then quenched by addition of water (150 mL). The resultant mixture was partitioned between EtOAc (200 mL) and H_2_O (100 mL). The aqueous phase was extracted with EtOAc (2 × 75 mL) and the combined organic phases washed successively with water (50 mL) and brine (50 mL), then dried over MgSO_4_ and filtered. The solvent was removed in vacuo. The residue was crystallized from isohexane/EtOAc to afford methyl 4-(3,5-dichloro-4-hydroxybenzamido)benzoate (**11**: R^2^ = H) (5.81 g, 84%): ^1^H NMR (400 MHz, DMSO‑*d*_6_) *δ* 11.06 (1H, s), 10.52 (1H, s), 8.06 (2H, s), 8.04–7.91 (4H, m), 3.88 (3H, s). *m*/*z* 338 (M−H)^−^ (ES^−^).

*Step (v)*: **Methyl 4-(4-(*tert*-butoxy)-3,5-dichlorobenzamido)benzoate**. A stirred suspension of methyl 4-(3,5-dichloro-4-hydroxybenzamido)benzoate (**11**: R^2^ = H) (100 mg, 294 µmol) in toluene (2 mL) was heated at 80 °C until homogenous. The resultant solution was treated with 1,1-di-*tert*-butoxy-*N*,*N*-dimethylmethanamine (141 µL, 588 µmol) and the mixture heated at 80 °C for 3 h, and then at room temperature for 18 h. Additional 1,1-di-*tert*-butoxy-*N*,*N*-dimethylmethanamine (141 µL, 588 µmol) was added and the mixture was heated at 80 °C for 5 h. The reaction mixture was cooled to room temperature and the solvent was removed in vacuo. The residue was diluted with water and extracted with Et_2_O. The organic layer was dried over MgSO_4_, filtered and concentrated in vacuo. The residue was partially purified by silica gel chromatography (12 g, 0–50% EtOAc in isohexane) to afford methyl 4-(4-(*tert*-butoxy)-3,5-dichlorobenzamido)benzoate (82 mg, 71%). The material was used in the next step without further purification.

*Step (vi)*: **4-(4-(*tert*-Butoxy)-3,5-dichlorobenzamido)benzoicacid (16)**. 4-(4-(*tert*-Butoxy)-3,5-dichlorobenzamido)benzoic acid **16** (39 mg, 51%) was prepared as a white solid from methyl 4-(4-(*tert*-butoxy)-3,5-dichlorobenzamido)benzoate (82 mg, 294 µmol) using a procedure essentially the same as in *step (ii)* for **15**: ^1^H NMR (400 MHz, DMSO‑*d*_6_) *δ* 12.79 (1H, s), 10.60 (1H, s), 8.06 (2H, s), 7.94 (2H, d, *J* = 8.1 Hz), 7.87 (2H d, *J* = 8.1 Hz), 1.49 (9H, s). *m*/*z* 380 [M−H]^−^ (ES^−^).

#### 5.1.3 4-(3,5-Dichloro-4-ethoxybenzamido)-2-methylbenzoic acid (18)

*Step (iii)*: **Methyl 4-(3,5-dichloro-4-ethoxybenzamido)-2-methylbenzoate** (**10: R^1^ = Et, R^2^ = Me)**. A solution of 3,5-dichloro-4-ethoxybenzoic acid (**7**: R^1^ = Et) (285 mg, 1.21 mmol) and DIPEA (1.05 mL, 6.05 mmol) in DMF (2.5 mL) was added to HATU (690 mg, 1.82 mmol) and the orange mixture was stirred for 5 min prior to the addition of methyl 4-amino-2-methylbenzoate (**8:** R = R^2^ = Me) (200 mg, 1.21 mmol) in DMF (1 mL). The resulting dark orange solution was stirred for 18 h. 2 M HCl (10 mL) was added and stirring continued for 10 min, and then the mixture was extracted with diethyl ether. The organic layer was washed with water (3 × 15 mL), dried over MgSO_4_, filtered and the solvent was evaporated in vacuo. The yellow residue was purified by silica gel chromatography (40 g, 0–100% EtOAc in isohexane) to afford methyl 4-(3,5-dichloro-4-ethoxybenzamido)-2-methylbenzoate (**10**: R^1^ = Et, R = R^2^ = Me) (267 mg, 56%): ^1^H NMR (400 MHz, CDCl_3_) *δ* 7.97 (d, *J* = 8.5 Hz, 1H), 7.81 (2H, s), 7.83–7.77 (1H, m), 7.59–7.48 (2H, m), 4.18 (2H, q, *J* = 7.0 Hz), 3.89 (3H, s), 2.63 (3H, s), 1.49 (3H, t, *J* = 7.0 Hz). *m*/*z* 380 (M−H)^−^ (ES^−^).

*Step (ii)*: **4-(3,5-Dichloro-4-ethoxybenzamido)-2-methylbenzoicacid (18)**. Lithium hydroxide (60 mg, 2.51 mmol) in water (1 mL) was added dropwise to a stirring solution of methyl 4-(3,5-dichloro-4-ethoxybenzamido)-2-methylbenzoate (**10**: R^1^ = Et, R = R^2^ = Me) (267 mg, 56%) (240 mg, 0.628 mmol) in THF (5 mL) and the resulting yellow solution was stirred for 5 days at room temperature. The solvent was evaporated in vacuo and dissolved in water (5 mL), then acidified with 2 M HCl. The resultant mixture was extracted with EtOAc. The organic layer was washed with water, dried over MgSO_4_ and filtered and pre-adsorbed on silica. Silica gel chromatography (40 g, 0–10% IPA in DCM) provided 4-(3,5-dichloro-4-ethoxybenzamido)-2-methylbenzoic acid **18** (52 mg, 22%) as a white solid: ^1^H NMR (400 MHz, DMSO‑*d*_6_) *δ* 12.66 (1H, s), 10.49 (1H, s), 8.08 (2H, s), 7.94–7.84 (1H, m), 7.75–7.65 (2H, m), 4.14 (2H, q, *J* = 7.0 Hz), 2.54 (3H, s), 1.40 (3H, t, *J* = 7.0 Hz). *m*/*z* 366 (M−H)^−^ (ES^−^).

The compound **58** was similarly prepared as **18**: see Supplementary data for experimental and spectroscopic details.

#### 5.1.4 4-(3,5-Dichloro-4-ethoxybenzamido)-2-hydroxybenzoic acid (**19**)

*Steps (vi) and (vii)*: ***tert*-Butyl 4-amino-2-methoxybenzoate** (**8**: R = *^t^*Bu, R^2^ = OMe). 1,1-di-*tert*-Butoxy-*N,N*-dimethylmethanamine (608 µL, 2.54 mmol) was added dropwise to a solution of 2-methoxy-4-nitrobenzoic acid **9** (250 mg, 1.27 mmol) in toluene (7.5 mL) at 80 °C. The reaction mixture was heated at 80 °C for 3 h, then a further quantity of 1,1-di-*tert*-butoxy-*N,N*-dimethylmethanamine (608 µL, 2.54 mmol) was added. The reaction mixture was heated at 80 °C for 16 h, then diluted with water (10 mL) and extracted with Et_2_O (3 × 10 mL). The combined organic phases were washed with brine (30 mL), dried over MgSO_4_, filtered and then concentrated in vacuo to afford *tert*-butyl 2-methoxy-4-nitrobenzoate (271 mg, 78%) as a pale yellow solid. The material was used in the next step without further purification. *tert*-Butyl 2-methoxy-4-nitrobenzoate (271 mg, 1.07 mmol) was dissolved in MeOH (270 mL) and passed through a Thales ‘H-cube’ cartridge (10% Pd/C) at a flow rate of 1 mL/min at 25 °C under full H_2_ mode. The solvent was removed in vacuo to afford *tert*-butyl 4-amino-2-methoxybenzoate (**8**: R = *^t^*Bu, R^2^ = OMe) (234 mg, 92%) as a pale yellow solid: ^1^H NMR (400 MHz, DMSO‑*d*_6_) *δ* 7.41 (1H, d, *J* = 8.5 Hz), 6.16 (1H, d, *J* = 2.0 Hz), 6.09 (1H, dd, *J* = 8.5, 2.0 Hz), 5.82 (2H, br s), 3.68 (3H, s), 1.45 (9H, s). *m*/*z* 222 [M−H]^−^ (ES^−^).

*Step (iii)*: ***tert*-Butyl 4-(3,5-dichloro-4-ethoxybenzamido)-2-methoxybenzoate** (**10: R^1^ = Et, R^2^ = OMe, R** = ***^t^*Bu)**. 3,5-Dichloro-4-ethoxybenzoic acid (**7**: R^1^ = Et) (75 mg, 0.32 mmol) in DCM (5 mL) was treated with oxalyl chloride (56 μL, 0.64 mmol) dropwise, followed by a drop of DMF. The reaction mixture was stirred at room temperature for 1 h, and then the solvent was removed in vacuo. The residue was dissolved in DCM (5 mL) and TEA (133 μL, 957 μmol) was added. The mixture was added to *tert*-butyl 4-amino-2-methoxybenzoate (**8**: R = *^t^*Bu, R^2^ = OMe) (71 mg, 0.32 mmol) and stirred at room temperature for 16 h. The mixture was sequentially washed with sat. aq. NaHCO_3_ (5 mL) and 1 M HCl (5 mL), and the organic phase was concentrated in vacuo. The residue was purified by silica gel chromatography (12 g, 0–100% EtOAc in isohexane) to afford *tert*-butyl 4-(3,5-dichloro-4-ethoxybenzamido)-2-methoxybenzoate (**10**: R^1^ = Et, R^2^ = OMe, R = *^t^*Bu**)** (59 mg, 42%) as a white solid: ^1^H NMR (400 MHz, DMSO‑*d*_6_) *δ* 10.51 (1H, s), 8.09 (2H, s), 7.63 (1H, d, *J* = 8.5 Hz), 7.60 (1H, d, *J* = 1.9 Hz), 7.43 (1H, dd, *J* = 8.5, 1.9 Hz), 4.15 (2H, q, *J* = 7.0 Hz), 3.81 (3H, s), 1.51 (9H, s), 1.41 (3H, t, *J* = 7.0 Hz). *m*/*z* 384 [M−*^t^*Bu+2H]^+^ (ES^+^).

*Step (viii)*: **4-(3,5-Dichloro-4-ethoxybenzamido)-2-hydroxybenzoicacid (19)**. A solution of *tert*-butyl 4-(3,5-dichloro-4-ethoxybenzamido)-2-methoxybenzoate (**10**: R^1^ = Et, R^2^ = OMe, R = *^t^*Bu) (55 mg, 0.13 mmol) in DCM (5 mL) was cooled to 0 °C and treated dropwise with a solution of 1 M boron trichloride in DCM (349 μL, 349 μmol). The reaction mixture was stirred at 0 °C for 1 h and then at room temperature for 2 h. The reaction mixture was cooled to 0 °C and water (0.5 mL) and sat. aq. NaHCO_3_ (2 mL) were added. The resulting white precipitate was collected by filtration and washed with water (2 mL). The solid was dried, then purified by capture and release on SAX, eluting with 5% AcOH in THF to afford 4-(3,5-dichloro-4-ethoxybenzamido)-2-hydroxybenzoic acid **19** (11 mg, 24%) as a white solid: ^1^H NMR (400 MHz, DMSO‑*d*_6_) *δ* 10.51 (1H, s), 8.06 (2H, s), 7.76 (1H, d, *J* = 8.7 Hz), 7.48 (1H, d, *J* = 2.0 Hz), 7.32–7.25 (1H, m), 4.14 (2H, q, *J* = 7.0 Hz), 1.91 (1H, s), 1.40 (3H, t, *J* = 7.0 Hz), 1.35 (1H, s). *m*/*z* 370 [M+H]^+^ (ES^+^), 368 [M−H]^−^ (ES^−^).

#### 5.1.5 4-(3,5-Dichloro-4-ethoxyphenylcarbamoyl)benzoic acid (26)

*Steps (i) and (ii)*: **4-(3,5-Dichloro-4-hydroxyphenylcarbamoyl)benzoicacid (25)**. A mixture of 4-(chlorocarbonyl)benzoic acid methyl ester **23** (600 mg, *ca.* 3.02 mmol) contaminated with 4-(methoxycarbonyl)benzoic acid **22** was suspended in DCM (5 mL) and cooled to 0 °C. The mixture was treated with oxalyl chloride (529 µL, 6.04 mmol) and DMF (1 drop). The resultant mixture was warmed to room temperature, stirred for 2 h, and then concentrated in vacuo. The residue was dissolved in DCM (3 mL) and a suspension of 4-amino-2,6-dichlorophenol **24** (511 mg, 2.9 mmol) in DCM (18 mL) was added. The resultant suspension was treated with DIPEA (1.58 mL, 9.06 mmol) and was stirred at room temperature for 16 h. The solvent was removed in vacuo and the residue partitioned between EtOAc/DCM and aqueous HCl (1 M). The layers were separated and the organic layer was washed with water and brine. The organic layer was dried over MgSO_4_, filtered and then the solvent evaporated in vacuo to afford a pale brown solid (930 mg), which was triturated in hot acetonitrile/methanol (9:1) and filtered. The precipitate and filtrate were recombined, the solvent was evaporated in vacuo and then the residue was dissolved in THF (40 mL). Water (10 mL) was added and the mixture treated with lithium hydroxide (340 mg, 14.2 mmol). The mixture was stirred for 16 h and then partitioned between EtOAc and aqueous HCl (1 M). The organic layer was washed successively with water (2 × 50 mL), brine, dried over MgSO_4_, filtered and then concentrated in vacuo to afford crude 4-(3,5-dichloro-4-hydroxyphenylcarbamoyl)benzoic acid **25** as a pale brown solid. This material was used in the subsequent reaction step without purification.

*Step (iii)*: **Ethyl 4-(3,5-dichloro-4-ethoxyphenylcarbamoyl)benzoate**. Crude 4-(3,5-dichloro-4-hydroxyphenylcarbamoyl)benzoic acid **25** (450 mg) was dissolved in DMF (15 mL) and treated with potassium carbonate (829 mg, 6.00 mmol) and iodoethane (436 µL, 5.4 mmol). The mixture was stirred at 65 °C for 16 h. Iodoethane (200 µL, 2.48 mmol) was added and the reaction mixture stirred at 70 °C for 3 h. The mixture was partitioned between EtOAc (150 mL) and aqueous HCl (100 mL, 1 M). The layers were separated and the organic layer was washed successively with saturated aqueous NaHCO_3_ and water. The organic layer was dried over MgSO_4_, filtered and the solvent evaporated in vacuo. The residue was purified by silica gel chromatography (10–25% EtOAc/isohexane) to afford ethyl 4-(3,5-dichloro-4-ethoxyphenylcarbamoyl)benzoate (500 mg, 75% over 2 steps) as a pale pink solid: *m*/*z* 380 (M−H)^+^ (ES^−^).

*Step (iv)*: **4-(3,5-Dichloro-4-ethoxyphenylcarbamoyl)benzoicacid (26)**. Ethyl 4-(3,5-dichloro-4-ethoxyphenylcarbamoyl)benzoate (109 mg, 285 µmol) in THF (5 mL) was treated with aqueous lithium hydroxide (1.43 mL, 1 M, 1.43 mmol) and the mixture was stirred at room temperature for 5 h. The reaction mixture was partitioned between EtOAc and aqueous HCl (1 M). The organic layer was separated and washed successively with water and brine. The organic layer was dried over MgSO_4_, filtered and then concentrated in vacuo to afford 4-(3,5-dichloro-4-ethoxyphenylcarbamoyl)benzoic acid **26** (89 mg, 88%) as a pale lilac solid: ^1^H NMR (400 MHz, DMSO‑*d*_6_) *δ* 13.30 (1H, s), 10.58 (1H, s), 8.13–7.99 (4H, m), 7.94 (2H, s), 4.04 (2H, q, *J* = 7.0 Hz), 1.37 (3H, t, *J* = 7.0 Hz). *m*/*z* 352 [M−H]^−^ (ES^−^).

#### 5.1.6 4-(4-Ethoxy-3,5-diisopropoxybenzamido)benzoic acid (**31**)

*Step (i)*: **Methyl 3,5-dihydroxy-4-ethoxybenzoate(28: R^1^ = Et)**. A mixture of methyl 3,4,5-trihydroxybenzoate **27** (5g, 27.2 mmol), iodoethane (2.194 mL, 27.2 mmol) and sodium hydrogen carbonate (9.12 g, 109 mmol) was stirred in *N,N*-dimethylformamide (50 mL) at 30 °C for 72 h. Water (50 mL) was added and the mixture was extracted with ethyl acetate (2 × 50 mL). The organic layer was then washed with water (50 mL), brine (50 mL), dried over magnesium sulfate, filtered and concentrated in vacuo. The product was then purified by silica gel chromatography (80 g, 0–20% hexane/ethyl acetate) to leave **28** (2.90 g, 50% yield). ^1^H NMR (400 MHz, CDCl_3_) *δ* 7.24 (2H, s), 5.68 (2H, s), 4.21 (2H, q, *J* = 7.1 Hz), 3.89 (3H, s), 1.42 (3H, t, *J* = 7.0 Hz).

*Step (ii)*: **3,5-Diisopropoxy-4-ethoxybenzoicacid (29: R^1^ = Et, R^2^ = R^3^ = iPr)**. Compound **28** (500 mg, 2.36 mmol) was combined with 2-bromopropane (885 μL, 9.43 mmol) and potassium carbonate (651 mg, 4.71 mmol) in *N,N*-dimethylformamide (5 mL). The resulting suspension was stirred at 50 °C for 48 h. Water (5 mL) was added and the mixture was extracted with ethyl acetate (2 × 5 mL). The organic layer was then washed with water (5 mL), brine (5 mL), dried over magnesium sulfate, filtered and concentrated in vacuo. The crude product was then purified by silica gel chromatography (40 g, 0–50% hexane/ethyl acetate) to leave methyl 3,5-diisopropoxy-4-ethoxybenzoate (530 mg, 76% yield). ^1^H NMR (400 MHz, CDCl_3_) *δ* 7.27 (2H, s), 4.61–4.55 (2H, m), 4.10 (2H, q, *J* = 7.1 Hz), 3.88 (3H, s), 1.38–1.33 (15H, m).

*Step (iii)*: Methyl 3,5-diisopropoxy-4-ethoxybenzoatewas converted to compound **(29: R^1^ = Et, R^2^ = R^3^ = iPr)** in 57% yield using lithium hydroxide in the procedure described for compound **15**. ^1^H NMR (400 MHz, CDCl_3_) *δ* 7.34 (2H, s), 4.61–4.55 (2H, m), 4.10 (2H, q, *J* = 7.1 Hz), 1.40–1.33 (15H, m).

*Step (iv)*: **4-(4-Ethoxy-3,5-diisopropoxybenzamido)benzoicacid (31)**. Compound **(29: R^1^ = Et, R^2^ = R^3^ = iPr)** and **30** were coupled and hydrolyzed to the title compound **31** (288 mg, 57% for final step) as a white solid, using the procedures described for the preparation of **15**. ^1^H NMR (400 MHz, CDCl_3_) *δ* 8.02 (2H, d, *J* = 8.7 Hz), 7.85 (1H, br s), 7.75 (2H, d, *J* = 8.8 Hz), 7.09 (2H, s), 4.66–4.58 (2H, m), 4.10 (2H, q, *J* = 7.1 Hz), 1.39–1.33 (15H, m). *m*/*z* 400 (M−H)^−^ (ES^−^), 402 (M+H)^+^ (ES^+^).

The compounds **32**–**34** were similarly prepared as **31**: see Supplementary data for experimental and spectroscopic details.

#### 5.1.7 4-[3-Chloro-4,5-bis(cyclopentyloxy)benzamido]benzoic acid (**42**)

*Step (i)*: **Methyl 3-chloro-4,5-dihydroxybenzoate (36)**. Tribromoborane (7.86 mL, 82 mmol) was added dropwise to a stirring mixture of 3-chloro-4-hydroxy-5-methoxybenzoic acid **35** (6.61 g, 32.6 mmol) in dichloromethane (50 mL) under nitrogen at 0 °C. The resulting orange mixture was stirred at the same temperature for 2 h then poured portion wise onto ice/brine (250 mL). The aqueous phase was extracted with ethyl acetate (2 × 150 mL) and the combined organic extracts were dried over magnesium sulfate and filtered. The solvent was removed in vacuo to give 3-chloro-4,5-dihydroxybenzoic acid (5.11 g, 79% yield). ^1^H NMR (400 MHz, DMSO‑*d*_6_) *δ* 12.69 (1H, br s), 10.14 (2H, br s), 7.35 (1H, d, *J* = 2.0 Hz), 7.32 (1H, d, *J* = 2.0 Hz). *m*/*z* 187 [M−H]^−^ (ES).

*Step (ii)*: A solution of 3-chloro-4,5-dihydroxybenzoic acid (3.16 g, 16.76 mmol) and chlorotrimethylsilane (6.36 mL, 50.3 mmol) in methanol (50 mL) was stirred at 50 °C, for 16 h, under an atmosphere of nitrogen. The solvent was removed in vacuo and the residue was partitioned between brine (75 mL) and ethyl acetate (75 mL). The organic layer was washed with brine (75 mL), dried over magnesium sulfate and filtered. The solvent was removed in vacuo to give methyl 3-chloro-4,5-dihydroxybenzoate **36** (3.26 g, 82% yield). ^1^H NMR (400 MHz, DMSO‑*d*_6_) *δ* 10.17 (2H, br s), 7.38 (1H, d, *J* = 2.0 Hz), 7.35 (1H, d, *J* = 2.0 Hz), 3.78 (3H, s). *m*/*z* 201 [M−H]^−^ (ES^−^).

*Step (iii)*: **3-Chloro-4,5-bis(cyclopentyloxy)benzoicacid** (**37, R = cyclopentyl**). A mixture of methyl 3-chloro-4,5-dihydroxybenzoate **36** (300 mg, 1.48 mmol), iodocyclopentane (558 µL, 4.44 mmol) and potassium carbonate (614 mg, 4.44 mmol) in DMF (10 mL) was stirred at 70 °C for 46 h. The reaction mixture was cooled to room temperature and then partitioned between 1 M hydrochloric acid (75 mL) and ethyl acetate (100 mL). The phases were separated and the organic phase was washed with brine (2 × 75 mL) then dried over magnesium sulfate and filtered. The solvent was removed in vacuo and the residue was purified by silica gel chromatography (40 g, 0–100% EtOAc and isohexane) to give methyl 3-chloro-4,5-bis(cyclopentyloxy)benzoate (427 mg, 85% yield). ^1^H NMR (400 MHz, CDCl_3_) *δ* 7.66 (1H, d, *J* = 2.0 Hz), 7.45 (1H, d, *J* = 2.0 Hz), 5.05–4.98 (1H, m), 4.87–4.83 (1H, m), 3.89 (3H, s), 1.95–1.55 (16H, m). *m*/*z* 339 [M+H]^+^ (ES^+^).

*Step (iv):* Methyl 3-chloro-4,5-bis(cyclopentyloxy)benzoate (400 mg, 1.18 mmol) was dissolved in a mixture of 1,4-dioxane (10 mL) and water (5 mL) and lithium hydroxide (226 mg, 9.44 mmol) was added. After stirring for 18 h at room temperature, the mixture was partitioned between 1 M hydrochloric acid (20 mL) and ethyl acetate (25 mL). The phases were separated and the organic phase was washed with water (20 mL) then dried over magnesium sulfate and filtered. The solvent was removed in vacuo to give the title compound (**37**: R = cyclopentyl) (380 mg, 99% yield). ^1^H NMR (400 MHz, DMSO‑*d*_6_) *δ* 13.07 (1H, br s), 7.52 (1H, d, *J* = 2.0 Hz), 7.45 (1H, d, *J* = 2.0 Hz), 4.97–4.91 (2H, m), 1.99–1.90 (2H, m), 1.70–1.57 (14H, m). *m*/*z* 323 [M−H]^−^ (ES^−^).

*Step (v) and (iv)*: **4-[3-Chloro-4,5-bis(cyclopentyloxy)benzamido]benzoicacid (42)**. A mixture of 3-chloro-4,5-bis(cyclopentyloxy)benzoic acid (**37**: R = cyclopentyl) and methyl 4-aminobenzoate (**38**: R^1^ = H) was converted to the methyl 4-[3-chloro-4,5-bis(cyclopentyloxy)benzamido]benzoate in 49% yield using the procedure in *step (iii)* described for compound **15**, ^1^H NMR (400 MHz, CDCl_3_) *δ* 8.06 (2H, d, *J* = 8.8H), 7.84 (1H, br s), 7.72 (2H, d, *J* = 8.8 Hz), 7.42–7.35 (2H, m), 5.08–4.98 (1H, m), 4.90–4.86 (1H, m), 3.92 (3H, s), 1.95–1.63 (16H, m). *m*/*z* 458 [M+H]^+^ (ES^+^), 456 [M−H]^−^ (ES^−^). Hydrolysis of methyl-4-[3-chloro-4,5-bis(cyclopentyloxy)benzamido]benzoate using the procedure in *step (iv)* described in the preparation of compound (**37**: R = cyclopentyl) gave 4-[3-Chloro-4,5-bis(cyclopentyloxy)benzamido]benzoic acid **42** in 72% yield as white solid. ^1^H NMR (400 MHz, DMSO‑*d*_6_) *δ* 12.74 (1H, br s), 10.45 (1H, s), 7.98–7.84 (4H, m), 7.69 (1H, d, *J* = 2.0 Hz), 7.52 (1H, d, *J* = 2.0 Hz), 5.01–4.95 (2H, m), 1.99–1.93 (2H, m), 1.73–1.48 (14H, m). *m*/*z* 442 [M−H]^−^ (ES^−^).

The compounds **39, 40, 43, 44** were similarly prepared as **42:** see Supplementary data for experimental and spectroscopic details.

#### 5.1.8 4-(3,4-Di-*tert*-butoxy-5-chlorobenzamido)benzoic acid (41)

*Step (ix)*: **3,4-Di-*tert*-butoxy-5-chlorobenzoicacid** (**37**: **R = *^t^*Bu**). *N,N*-Dimethylformamide di-*tert*-butyl acetal (5.92 mL, 24.7 mmol) was added to a solution of methyl 3-chloro-4,5-dihydroxybenzoate **36** (500 mg, 2.47 mmol) in toluene (10 mL) and the reaction mixture was stirred at RT under nitrogen for 21 h. The solvent was removed in vacuo and the residue was purified by silica gel chromatography (40 g, 0–20% EtOAc in iso-hexane) to give the *bis*-alkylated intermediate, which was dissolved in 1,4-dioxane/water (20 mL, 1:1) and treated with lithium hydroxide (591 mg, 24.7 mmol). The mixture was stirred 18 h at room temperature. The mixture was poured into 10% aqueous citric acid (100 mL) and the precipitate was collected by filtration. The solid was washed with water and dried to give 3,4-di-*tert*-butoxy-5-chlorobenzoic acid (**37**: R = *^t^*Bu) (534 mg, 70%). ^1^H NMR (400 MHz, DMSO‑*d*_6_) *δ* 13.13 (1H, br s), 7.67 (1H, s), 7.53 (1H, s), 1.39 (9H, s), 1.32 (9H, s). *m*/*z* 299 [M−H]^−^ (ES^−^).

*Step (x)*: 4-(3,4-Di-*tert*-butoxy-5-chlorobenzamido)benzoic acid (**41**).

A mixture of 3,4-di-*tert*-butoxy-5-chlorobenzoic acid (**37**: R = *^t^*Bu) (250 mg, 0.831 mmol) and methyl 4-aminobenzoate (**38**: R^1^ = H) was converted to themethyl 4-(3,4-di-*tert*-butoxy-5-chlorobenzamido)benzoate (185 mg, 50%) using the procedure described for compound **18**. ^1^H NMR (400 MHz, DMSO‑*d*_6_) δ:10.54 (1H, s), 7.96 (2H, d), 7.91 (2H, d), 7.86 (1H, d), 7.60 (1H, d), 3.84 (3H, s), 1.41 (9H, s), 1.32 (9H, s). *m*/*z* 432 [M−H]^−^ (ES^−^). Hydrolysis of methyl 4-(3,4-di-*tert*-butoxy-5-chlorobenzamido)benzoate (175 mg, 0.403 mmol) using the procedure described in the preparation of compound (**37**: R = cyclopentyl) *step (vii)*. gave 4-(3,4-di-*tert*-butoxy-5-chlorobenzamido)benzoic acid **41** (110 mg, 64%) as a white solid: ^1^H NMR (400 MHz, DMSO‑*d*_6_) *δ* 12.78 (1H, br s). 10.49 (1H, s), 7.97–7.86 (3H, m), 7.85 (2H, d, *J* = 2.3 Hz), 7.59 (1H, d, *J* = 2.2 Hz), 1.40 (9H, s), 1.34 (9H, s). *m*/*z* 418 [M−H]^−^ (ES^−^).

The compound **45** was similarly prepared as **41**: see Supplementary data for experimental and spectroscopic details.

#### 5.1.9 4-(3-Chloro-4-ethoxy-5-isopropoxybenzamido)-2-methylbenzoic acid (**56**)

*Step (iii)*: **Methyl 4-benzyloxy-3-chloro-5-hydroxybenzoate (46)**. Methyl 3-chloro-4,5-dihydroxybenzoate **36** (14.19 g, 70 mmol) was dissolved in *N,N*-dimethylformamide (210 mL) and treated with potassium carbonate (8.71 g, 63 mmol). After stirring for 5 min, benzyl bromide (8.32 mL, 70 mmol) was added and the mixture was heated to 60 °C for 0.75 h. The reaction mixture was diluted with diethyl ether (500 mL) and washed successively with 1 M hydrochloric acid (500 mL) and with brine (2 × 500 mL). The aqueous phase was re-extracted with diethyl ether (500 mL) and the combined organic layers were washed with brine (2 × 500 mL) and dried with magnesium sulfate. Filtration and evaporation left the crude product which was purified by silica gel chromatography (330 g, 0–100% ethyl acetate/isohexane) to leave methyl 4-(benzyloxy)-3-chloro-5-hydroxybenzoate **46** as an off-white solid (9.84 g, 48% yield). ^1^H NMR (400 MHz, DMSO‑*d*_6_) *δ* 10.50 (1H, s), 7.57–7.53 (2H, m), 7.52 (1H, d, *J* = 2.1 Hz), 7.47 (1H, d, *J* = 2.1 Hz), 7.46–7.37 (3H, m), 5.14 (2H, s), 3.82 (3H, s). (*m*/*z* 293.3 [M+H]^+^ (ES^+^), 291.2 [M−H]^−^ (ES^−^).

*Step (iv)*: **Methyl 3-chloro-4-benzyloxy-5-isopropoxybenzoate** (**47**: R^2^ = iPr). Methyl 4-(benzyloxy)-3-chloro-5-hydroxybenzoate **46** (7.5 g, 25.6 mmol) was combined with potassium carbonate (7.08 g, 51.2 mmol) in *N,N*-dimethylformamide (25 mL). The mixture was stirred at RT for 5 min. 2-Bromopropane (4.81 mL, 51.2 mmol) was added and the mixture stirred at 60 °C for 2 h. Water (25 mL) was added and the mixture was extracted with ethyl acetate (3 × 50 mL). The combined organic phase was washed with brine (2 × 50 mL) and then dried over magnesium sulfate, filtered and concentrated in vacuo to leave a crude mixture which was purified by silica gel chromatography (120 g, 0–100% ethyl acetate/isohexane) to afford methyl 3-chloro-4-benzyloxy-5-isopropoxybenzoate (**47**: R^2^ = iPr), as a clear oil (4.91 g, 57% yield). ^1^H NMR (400 MHz, DMSO‑*d*_6_) *δ* 7.68 (1H, d, *J* = 2.0 Hz), 7.56–7.47 (3H, m), 7.43–7.28 (3H, m), 5.12 (2H, s), 4.77–4.72 (1H, m), 3.90 (3H, s), 1.38 (6H, d, *J* = 6.1 Hz). *m*/*z* 335 [M+H]^+^ (ES^+^).

**3-Chloro-4-ethoxy5-isopropoxybenzoicacid** (**48**: R^2^ = iPr, R^3^ = Et). Steps (v), (vi) and (vii).

*Step (v)*: **Methyl 3-chloro-4-hydroxy-5-isopropoxybenzoate**. Methyl 3-chloro-4-(benzyloxy)-5-isopropoxybenzoate (**47**: R^2^ = iPr) (4.91 g, 14.7 mmol) was dissolved in a mixture of methanol (160 mL), dichloromethane (16 mL) and acetic acid (0.16 mL) and the solution was passed through a Thales ‘H-cube’ cartridge (10% Pd/C) at a flow rate of 1 mL/min at 25 °C under an atmosphere of hydrogen (full H_2_ mode). The solvents were removed in vacuo to afford methyl 3-chloro-4-hydroxy-5-isopropoxybenzoate (3.42 g, 85%). ^1^H NMR (400 MHz, DMSO‑*d*_6_) *δ* 10.07 (1H, s), 7.53 (1H, d, *J* = 2.0 Hz), 7.43 (1H, d, *J* = 2.0 Hz), 4.70–4.63 (1H, m), 3.82 (3H, s), 1.30 (6H, d, *J* = 6.0 Hz). *m*/*z* 245 [M+H]^+^ (ES^+^), 243 [M−H]^−^ (ES^−^).

*Step (vi)*: **Methyl 3-chloro-4-ethoxy-5-isopropoxybenzoate**. Methyl 3-chloro-4-hydroxy-5-isopropoxybenzoate (3.42 g, 14 mmol) was combined with potassium carbonate (3.86 g, 28 mmol) in *N,N*-dimethylformamide (5 mL) and the mixture heated at 60 °C for 10 min. Iodoethane (2.26 mL, 28 mmol) was added dropwise whereupon the mixture was stirred at 40 °C for 3 h. A further aliquot of iodoethane was added and heating and stirring was continued for 16 h. Water (50 mL) was added and the mixture was extracted with ethyl acetate (3 × 100 mL). The combined organic phase was washed with brine (3 × 50 mL) and then dried over magnesium sulfate, filtered and concentrated in vacuo to leave a crude mixture which was purified by silica gel chromatography (120 g, 0–100% ethyl acetate/isohexane) to afford methyl 3-chloro-4-ethoxy-5-isopropoxybenzoate as a white solid (3.17 g, 82% yield). ^1^H NMR (400 MHz, DMSO‑*d*_6_) *δ* 7.67 (1H, d, *J* = 2.0 Hz), 7.48 (1H, d, *J* = 2.5 Hz), 4.66–4.59 (1H, m), 4.16 (2H, q, *J* = 7.1 Hz), 3.90 (3H, s), 1.40 (3H, t, *J* = 7.0 Hz), 1.37 (6H, d, *J* = 6.0 Hz). *m*/*z* 245 [M+H]^+^ (ES^+^), 243 [M−H]^−^ (ES^−^).

*Step (vii)*: **3-Chloro-4-ethoxy5-isopropoxybenzoicacid** (**48**: R^2^ = iPr, R^3^ = Et). Methyl 3-chloro-4-ethoxy-5-isopropoxybenzoate (3.17 g, 11.6 mmol) was dissolved in tetrahydrofuran (226 mL) and treated with 1 M aqueous lithium hydroxide solution (23.25 mL, 23.25 mmol). Methanol (5 mL) was added so that a solution formed and this was heated at 40 °C for 1 h. After stirring for a further 16 h at room temperature, the reaction mixture was acidified with 1 M hydrochloric acid and extracted with diethyl ether (3 × 100 mL). The organic layer was dried over magnesium sulfate, filtered and concentrated in vacuo to leave 3-chloro-4-ethoxy 5-isopropoxybenzoic acid(**48**: R^2^ = iPr, *R*^3^ = Et) as a white solid (2.83 g, 94% yield). ^1^H NMR (400 MHz, DMSO‑*d*_6_) *δ* 13.21 (1H, s), 7.54 (1H, d, *J* = 1.9 Hz), 7.48 (1H, d *J* = 1.9 Hz,), 4.74–4.78 (1H, m), 4.11 (2H, q, *J* = 7.0 Hz), 1.37–1.21 (9H, m). *m*/*z* 257 [M−H]^−^ (ES^−^).

#### 5.1.10 4-(3-Chloro-4-ethoxy-5-isopropoxybenzamido)-2-methylbenzoic acid (**56**)

*Step (viii)*: **Methyl 4-(3-chloro-4-ethoxy-5-isopropoxybenzamido)-2-methylbenzoate**. A suspension of 3-chloro-4-ethoxy 5-isopropoxybenzoic acid (**48**: R^2^ = iPr, *R*^3^ = Et) (2.82 g, 10.9 mmol) and methyl 4-amino-2-methylbenzoate (**38**: R^1^ = Me) (2.16 g, 13.1 mmol) in ethyl acetate (33 mL) was treated with triethylamine (4.56 mL, 32.7 mmol) followed by T3P (50 wt% in ethyl acetate) (17.34 mL, 27.3 mmol) and the mixture was heated at 60 °C for 4 h and allowed to cool to room temperature for 16 h. The reaction mixture was stirred vigorously with an aqueous solution of sodium hydrogencarbonate (50 mL) for 10 min and separated. The aqueous layer was extracted with dichloromethane (3 × 100 mL) and the combined organic phases were dried (magnesium sulfate), filtered concentrated in vacuo and the residue purified by silica gel chromatography (40 g, 0:50:50 to 20:40:40 ethyl acetate:dichloromethane:isohexane) to produce methyl 4-(3-chloro-4-ethoxy-5-isopropoxybenzamido)-2-methylbenzoate as a beige solid (3.24 g, 70% yield). ^1^H NMR (400 MHz, CDCl_3_) *δ* 8.0 (1H, d, *J* = 8.4 Hz), 7.8 (1H, s), 7.59–7.50 (2H, m), 7.43–7.36 (2H, m), 4.69–4.73 (1H, m), 4.18 (2H, q, *J* = 7.1 Hz), 3.92 (3H, s), 2.6 (3H, s), 1.46–1.37 (9H, m). *m*/*z* 406 [M+H]^+^ (ES^+^), 404 [M−H]^−^ (ES^−^).

*Step (ix)*:1 M Lithium hydroxide solution (15.97 mL, 15.97 mmol) was added to a solution of methyl 4-(3-chloro-4-ethoxy-5-isopropoxybenzamido)-2-methylbenzoate (3.24 g, 7.98 mmol) in tetrahydrofuran (32 mL). Methanol (5 mL) was added and the mixture stirred at 40 °C for 16 h. A further aliquot of lithium hydroxide solution (7.98 mL, 7.98 mmol) in methanol (5 mL) was added and stirring at 40 °C was continued for 3 h. The reaction mixture was partitioned between water (50 mL) and diethyl ether (100 mL). The layers were separated and the aqueous layer was acidified with 1 M hydrochloric acid solution. A precipitate evolved which was filtered and washed with water (3 × 10 mL) and diethyl ether (3 × 10 mL). After drying, this left 4-(3-chloro-4-ethoxy-5-isopropoxybenzamido)-2-methylbenzoic acid **56** as a white solid (2.55 g, 81%). Recrystallisation from dioxane/water (82:18) gave white crystals mp 186 °C. ^1^H NMR (400 MHz, DMSO‑*d*_6_) *δ* 12.64 (1H, br s), 10.34 (1H, s), 7.86 (1H, d, *J* = 8.5 Hz,), 7.74–7.63 (3H, m), 7.55 (1H, d, *J* = 2.0 Hz), 4.79–4.73 (1H, m), 4.10 (2H, q, *J* = 7.1 Hz), 2.52 (3H, s), 1.37–1.26 (9H, m). ^13^C NMR (101 MHz, DMSO‑*d*_6_) *δ* 168.5, 164.4, 151.9, 148.0, 142.4, 140.9, 132.0, 130.9, 127.9, 125.5, 123.0, 121.4, 117.7, 114.2, 71.6, 69.3, 22.3, 22.2, 15.9. *m*/*z* 392 [M+H]^+^ (ES^+^), 390 [M−H]^−^ (ES^−^). HRMS: C_20_H_23_ClNO_5_ requires (M+H)^+^ 392.1265, found 392.1249 (error −4.0 ppm).

The compounds **49**–**55, 57** and **59** were similarly prepared as **56**: see Supplementary data for experimental and spectroscopic details.

### 5.2 Biological and ADME assays

#### 5.2.1 Transactivation assays for mouse RAR alpha, beta and gamma receptors

Transcriptional transactivation assays have been performed with *gal4* fusion receptor constructs, created using each of the mouse RAR ligand-binding domains, co-transfected with the pFR-luc (Stratagene) reporter construct in COS-7 cells. Thus, transfected cells will constitutively express the gal4-RAR fusion protein which in turn may be transactivated by ATRA to induce the expression of the *luciferase* that is driven by a gal4UAS. Briefly, on day one, 96 well plates were seeded with 8000 cells per well then left to recover overnight. On day two, the cells were co-transfected with 100 ng of reporter plasmid and 10 ng of the appropriate receptor plasmid per well using lipofectamine (Invitrogen). On day three, the lipofectamine containing media was replaced by a DMEM without phenol red, followed by the addition of novel compounds dissolved in 1 µL of DMSO to each well’s 100 µL total volume. Finally, on day four, the cells were lysed and their luciferase substrate was provided by the BrightGlo reagent (Promega), the plates were then read on the MicroBeta TriLux (Perkin Elmer). In each experiment, an 8 point dose response curve of ATRA was run in duplicate, and the various compounds tested were compared to these values.

#### 5.2.2 FlashPlate® Scintillation Proximity Binding Assay (SPA)

In the FlashPlate® Scintillation Proximity Assay (SPA) wells of a 96-well plate are coated with scintillant and capture antibody (or similar) for tagged proteins. This requires just 100 ng of RAR proteins and 2 nM [^3^H]-retinoic acid per well. This enables competition of specifically bound [^3^H]-retinoic acid by unlabelled retinoid compounds. As only radioligand specifically bound to the captured protein is sufficiently close to the scintillant to produce a signal, separation of bound and free radioactivity is not required. Binding of the tritiated retinoid to biotinylated RARα is specific, saturable, time dependent and reversible. We have successfully applied our assay to a screen of known retinoid standards and novel compounds and it is both rapid and reproducible (see Supplementary data file for details).

#### 5.2.3 Intrinsic clearance Cl_int_

In this in vitro model of hepatic clearance mouse or human liver microsomes were incubated with the test compound at 37 °C in the presence of the co-factor, NADPH, which initiates the reaction. The reaction is terminated by the addition of methanol. Following centrifugation, the supernatant is analyzed on the LC-MS/MS. The disappearance of the test compound is monitored over a 45 min time period. The data is the mean on 5 separate experiments. SEM is less than 10% of the mean values.

The ln peak area ratio (compound peak area/internal standard peak area) is plotted against time and the gradient of the line determined.

The elimination rate constant (k) = (−gradient), the Half life (t_1/2_)(min) = 0.693/k and V(*µ*L/mg) = volume of incubation (*µ*L)/protein in the incubation (mg).

Intrinsic Clearance = (**CL_int_**)(*µ*L/min/mg/protein) = V × 0.693/k. (see Supplementary data file for details).

#### 5.2.4 PK studies in rats

Test compounds were administered orally and intravenously to groups of 4 male Sprague-Dawley rats. Oral dosing solutions of each Test Item were prepared at a concentration of 1 mg/mL in 8% ethanol and 92% PEG-400. The Test Items were orally administered at a dose of 10 mg/kg and a dosing volume of 10 mL/kg. Intravenous dosing solutions of each Test Item were prepared at a concentration of 0.25 mg/mL in 8% ethanol, 92% PEG-400. The Test Items were intravenously administered at a dose of 0.5 mg/kg and a dosing volume of 2 mL/kg. Approximately eight blood samples were collected from each animal at appropriate intervals up to 6 h post dosing for the iv groups and up to 24 h for the oral groups. Whole blood concentrations of the Test Items were measured using LC-MS/MS and selected pharmacokinetic parameters calculated using Pharsight WinNonLin software. For furthers details see Supplementary data file.
