# Supplementary material for: Design and synthesis of a potent, highly selective, orally bioavailable, retinoic acid receptor alpha agonist
Source: Bioorg Med Chem. 2018 Feb 15;26(4):798–814. doi: 10.1016/j.bmc.2017.12.015 (PMC5823845; doi:10.1016/j.bmc.2017.12.015)
Supplement: Supplementary data [file mmc1.doc]

Supplementary data

**Design and Synthesis of a Potent, Highly Selective, Orally Bioavailable, Retinoic Acid Receptor Alpha Agonist.**

**Earl Clarke,^†^ Christopher I. Jarvis,^†^ Maria B. Goncalves,^†^ S. Barret Kalindjian,^†^ David R. Adams,^‡^ Jane T. Brown,^‡^ Jason J. Shiers,^‡^ David M. A. Taddei,^‡^ Elodie Ravier,^‡^ Stephanie Barlow,^‡^ Iain Miller,^‡^ Vanessa Smith,^‡^ Alan D. Borthwick,^δ^* and Jonathan P. T. Corcoran.^†^***

**Contents page**

Experimental and spectroscopic details for similarly prepared compounds 1

Biological assays 6

Production of biotin-RAR-ligand binding domain 6

Transient Cell Transfections for Transactivation Studies 6

Dual Luciferase Assays 7

Data Analysis 7

The FlashPlate® Scintillation Proximity Binding Assay 8

Invitrogen Transactivation RAR assays 8

In Vivo Pharmacokinetics and Oral Bioavailability 8

ADME assays 9

Cellular Toxicity assays 10

Genetic Toxicity assays 10

In Vitro Pharmacology: Binding and Enzyme Assays: 11

Virtual Screening and Molecular Modelling 11

References. 15

**Experimental and spectroscopic details for similarly prepared compounds.**

This includes similarly prepared compounds **12 – 14**, **17, 20,** **21, 32 – 34, 39, 40, 43** **–** **45,** **49** **–** **55,** **57** **–** **59, 63** and **64.**

The compounds **12 – 14**, **17, 20,** **21, 63** and **64** were similarly prepared as **15**.

**4-(3,5-Dichloro-4-propoxybenzamido)benzoic acid** (**12**) (34 mg, 71% for final step) was prepared as a white solid in essentially the same manner as in *steps (iii) and (ii)* for **15** except that 3,5-dichloro-4-propoxybenzoic acid was used instead of 3,5-dichloro-4-(cyclopentyloxy)-benzoic acid in *step (iii)*: ^1^H NMR (400 MHz, DMSO-*d*_6_) *δ* 12.77 (1H, s), 10.59 (1H. s), 8.08 (2H, s), 7.98 – 7.90 (2H, m), 7.92 – 7.83 (2H, m), 4.03 (2H, t, *J* = 6.4 Hz), 1.84 – 1.77 (2H, m), 1.04 (3H, t, *J* = 7.4 Hz). m/z 366 (M-H)^-^ (ES^-^).

**4-(4-Butoxy-3,5-dichlorobenzamido)benzoic acid** (**13)** (42 mg, 67% for final step) was prepared as a white solid from methyl 4-(3,5-dichloro-4-hydroxybenzamido)benzoate (**11:** R^2^ = H) in essentially the same manner as in *steps (v) and (ii)* for **15** except that 1-bromobutane was used instead of bromocyclopentane: ^1^H NMR (400 MHz, CD_3_OD) *δ* 8.07 – 7.99 (4H, m), 7.89 – 7.80 (2H, m), 4.12 (2H, t, *J* = 6.4 Hz), 1.89**–**1.82 (2H, m) 1.65**–**1.56 (2H, m), 1.03 (3H, t, *J* = 7.4 Hz). m/z 382.1/383.9 (M+H)^+^ (ES^+^), 380.0/382.1 (M-H)^-^ (ES^-^).

**4-(3,5-Dichloro-4-isopropoxybenzamido)benzoic acid** (**14)** (48.5 mg, 53% for final step) was prepared as a white solid in essentially the same manner as **15** except that isopropyl bromide was used instead of cyclopentyl bromide in *step (i)*: ^1^H NMR (400 MHz, CD_3_OD) *δ* 8.06 – 7.98 (4H, m), 7.87 – 7.77 (2H, m), 4.80 – 4.74 (1H, m), 1.38 (6H, d, *J* = 6.2 Hz). m/z 366 (M-H)^-^ (ES^-^), 368 (M+H)^+^ (ES^+^).

**4-(3,5-Dibromo-4-ethoxybenzamido)benzoic acid (63)** (42 mg, 87% for final step) was prepared as a white solid in essentially the same manner as in *steps (iii) and (iv)* for **15**, except that 3,5-dibromo-4-ethoxybenzoic acid (prepared in 2 steps from methyl 3,5-dibromo-4-hydroxybenzoate by sequential treatment with ethyl iodide and base and then lithium hydroxide) was used instead of 3,5-dichloro-4-(cyclopentyloxy)benzoic acid in *step (iii)*: ^1^H NMR (400 MHz, DMSO-*d*_6_) *δ* 12.77 (1H, s), 10.59 (1H, s), 8.24 (2H, s), 7.98 – 7.83 (4H, m), 4.10 (2H, q, *J* = 7.0 Hz), 1.43 (3H, t, *J* = 7.1 Hz). m/z 442 (M-H)^-^ (ES^-^).

**4-(3,5-Di-*tert*-Butyl-4-ethoxybenzamido)benzoic acid (64**) (40 mg, 57% for final step) was prepared as a white solid in essentially the same manner as in *steps (iii) and (iv)* for **15**, except that 3,5-di-*tert*-butyl-4-ethoxybenzoic acid (prepared in 2 steps from 3,5-di-*tert*-butyl-4-hydroxy benzoic acid methyl ester by sequential treatment with ethyl iodide and base and then lithium hydroxide) was used instead of 3,5-dichloro-4-(cyclopentyloxy)benzoic acid in *step (iii)*: ^1^H NMR (400 MHz, DMSO-d_6_) *δ* 12.70 (1H, br s), 10.36 (1H, s), 7.97 – 7.89 (2H, m), 7.90 – 7.83 (2H, m), 7.80 (2H, s), 3.75 (2H, q, *J* = 6.9 Hz), 1.43 (18H, s), 1.38 (3H, t, *J* = 7.0 Hz). m/z 398 [M+H]^+^ (ES^+^), 396 [M-H]^-^ (ES^-^).

**4-(3,5-Dichloro-4-ethoxybenzamido)-2-fluorobenzoic acid (17)** (380 mg, 48% for final step) was prepared as a white solid in essentially the same manner as for **15** except that ethyl iodide was used instead of cyclopentyl bromide in *step (i)* and methyl 4-amino-2-fluorobenzoate (**8:**R = Me R^2^ = F) (prepared by the action of hydrogen and10% Pd/C on methyl 2-fluoro-4-nitrobenzoate) was used instead of methyl 4-aminobenzoate (**8:**R = Me R^2^ =H) in *step* *(iii)*: ^1^H NMR (400 MHz, MeOH-d_4_) *δ* 8.01 (2H, s), 7.92 (1H, t, *J* = 8.5 Hz), 7.79 (1H, dd, *J* = 13.3, 2.0 Hz), 7.57 – 7.49 (1H, m), 4.18 (2H, q, *J* = 7.0 Hz), 1.46 (3H, t, *J* = 7.0 Hz,). m/z 370 (M-H)^-^ (ES^-^), 372 (M+H)^+^ (ES^+^).

**2-Chloro-4-(3,5-dichloro-4-ethoxybenzamido)benzoic acid (20)** (39 mg, 39% for the final step) was prepared as a white solid in essentially the same manner as in *steps (iii) and (ii)* for  **15**, except that 3,5-dichloro-4-ethoxybenzoic acid (**7:** R^1^ = Et) was used instead of 3,5-dichloro-4-(cyclopentyloxy)benzoic acid and methyl 4-amino-2-chlorobenzoate (**8:**R = Me R^2^ = Cl) was used instead of methyl 4-aminobenzoate (**8:**R = Me R^2^ = H) in *step (iii)*: ^1^H NMR (400 MHz, DMSO‑*d*_6_) *δ* 13.18 (1H, s), 10.65 (1H, s), 8.07 (2H, s), 8.00 (1H, d, *J* = 2.0 Hz), 7.94 – 7.85 (1H, m), 7.78 (1H, dd, *J* = 8.6, 2.1 Hz), 4.13 (2H, q, *J* = 7.0 Hz), 1.39 (3H, t, *J* = 7.0 Hz). m/z 386 (M-H)^-^ (ES^‑^).

**4-(3,5-Dichloro-4-ethoxybenzamido)-2-(trifluoromethyl)benzoic acid (21)** (12 mg, 10% for final step) was prepared as a white solid in essentially the same manner as for **15**, except that ethyl iodide was used instead of cyclopentyl bromide in *step (i)* and methyl 4-amino-2-(trifluoromethyl)benzoate (**8:**R = Me R^2^ = CF_3_) (prepared from 4-amino-2-(trifluoromethyl)benzoic acid by reaction with MeOH and TMSCl) was used instead of methyl 4-aminobenzoate in (**8:**R = Me R^2^ = H) *step (iii)*: ^1^H NMR (400 MHz, DMSO‑*d*_6_) *δ* 10.77 (1H, s), 8.26 (1H, s), 8.14 (1H, dd, *J* = 8.5, 2.1 Hz ), 8.10 (2H, s), 7.93 – 7.86 (1H, m), 7.15 (1H, br s), 4.14 (2H, q, *J* = 7.0 Hz), 1.40 (3H, t, *J* = 7.0 Hz). m/z 422 [M+H]^+^ (ES^+^); 420 [M-H]^-^ (ES^-^).

The compounds **32 – 34** were similarly prepared as **31**.

**4-(3,5-Bis(cyclopentyloxy)-4-ethoxybenzamido)benzoic acid** (**32**) (75 mg, 55% for final step) was prepared as a white solid from methyl 3,4,5-trihydroxybenzoate in essentially the same manner as **31** except that cyclopentyl bromide was used instead of 2-bromopropane in *step (ii)*: ^1^H NMR (400 MHz, CDCl_3_) *δ* 8.16 – 8.08 (2H, m), 7.83 (1H, s), 7.80 – 7.72 (2H, m), 7.04 (2H, s), 4.89 – 4.85 (2H, m), 4.06 (2H, q, *J* = 7.0 Hz), 2.00**–**1.50 (16H, m), 1.35 (3H, t, *J* = 7.0 Hz). m/z 454 [M+H]^+^ (ES^+^), 452 [M-H]^-^ (ES^‑^).

**4-(3,4,5-Triisopropoxybenzamido)benzoic acid** (**33**) (194 mg, 91% for final step) was prepared as a white solid in essentially the same manner as **31** except that 3,4,5-triisopropoxybenzoic acid (**29 :** R^1^ = R^2^ = R^3^ = iPr) (prepared in 2 steps from methyl 3,4,5-trihydroxybenzoate by sequential reaction with 2-bromopropane and base and then lithium hydroxide) was used instead of 3,5-Diisopropoxy-4-ethoxybenzoic acid (**29 :** R^1^ = Et, R^2^ = R^3^ = iPr) in *step (iv)*: ^1^H NMR (400 MHz, CDCl_3_) *δ* 8.16 – 8.09 (2H, m), 7.87 (1H, s), 7.80 – 7.71 (2H, m), 7.08 (2H, s), 4.67 – 4.61 (2H, m), 4.49 – 4.42 (1H, m), 1.36 (12H, d, *J* = 6.1 Hz), 1.32 (6H, d, *J* = 6.2 Hz). m/z 416 [M+H]^+^ (ES^+^), 414 [M-H]^-^ (ES^-^).

**4-(3,4-Diethoxy-5-isopropoxybenzamido)benzoic acid** (**34**) (5 mg, 15% for final step) was prepared as a white solid in essentially the same manner as in *steps (iii) and (iv)* for **31** except that 3,4-diethoxy-5-diisopropoxybenzoic acid (prepared in 3 steps from methyl 3,4,5-trihydroxybenzoate by sequential treatment with ethyl iodide and base, isopropyl bromide and base and then lithium hydroxide, the product being a by-product of the preparation of 4-ethoxy-3,5-diisopropoxybenzoic acid shown in the synthesis of **31** was used instead of 3,5-diisopropoxy-4-ethoxybenzoic acid in *step (iv)*: ^1^H NMR (400 MHz, CDCl_3_) *δ* 8.16 – 8.08 (2H, m), 7.80 – 7.71 (2H, m), 7.12 – 7.05 (2H, m), 4.66 – 4.60 (1H, m), 4.17 – 4.11 (4H, m), 1.47 (3H, t, *J* = 7.0 Hz), 1.40-1.36 (9H, m). m/z 386 (M-H)^-^ (ES^-^), 388 (M+H)^+^ (ES^+^).

The compounds **39, 40, 43, 44** were similarly prepared as **42.**

**4-(3-Chloro-4,5-diisopropoxybenzamido)benzoic acid** (**39**) (134 mg, 74% for final step) was prepared as a white solid in essentially the same manner as **42** except isopropyl bromide was used instead of cyclopentyl iodide and the reaction performed at 80 °C in *step (ix)*: ^1^H NMR (400 MHz, DMSO‑*d*_6_) *δ* 12.78 (1H, br s), 10.46 (1H, s), 7.94 (2H, d, *J* = 8.8 Hz), 7.87 (2H, d, *J* = 8.9 Hz), 7.68 (1H, d, *J* = 2.0 Hz), 7.54 (1H, d, *J* = 2.0 Hz), 4.79 – 4.73 (1H, m), 4.62 – 4.56 (1H, m), 1.33 (6H, d, *J* = 6.0 Hz), 1.28 (6H, d, *J* = 6.2 Hz). m/z 390 [M-H]^-^ (ES^-^).

**4-(3-Chloro-4,5-di(cyclobutyloxy)benzamido)benzoic acid** (**40**) (137 mg, 54% for final step) was prepared as a white solid in essentially the same manner as **42** except cyclobutyl bromide was used instead of cyclopentyl iodide and the reaction performed at 80 °C in *step (ix)*: ^1^H NMR (400 MHz, DMSO‑*d*_6_) *δ* 12.76 (1H, br s), 10.46 (1H, s), 7.98**–**7.83 (4H, m), 7.70 (1H, d, *J* = 2.0 Hz), 7.34 (1H, d, *J* = 2.0 Hz), 4.88 – 4.80 (1H, m), 4.76 – 4.67 (1H, m), 2.49**–**2.42 (2H, m), 2.36**–**2.01 (6H, m), 1.92**–**1.61 (3H, m), 1.57**–**1.37 (1H, m). m/z 414 [M-H]^-^ (ES^-^).

**4-(3-Chloro-4,5-diisopropoxybenzamido)-2-fluorobenzoic acid** (**43**) (207 mg, 93% for final step) was prepared as a white solid in essentially the same manner as **42** except isopropyl bromide was used instead of cyclopentyl iodide and the reaction performed at 80 °C in *step (ix)* and methyl 4-amino-2-fluorobenzoate (**38,** R^1^ = F) was used instead of methyl 4-aminobenzoate (**38,** R^1^ = H): ^1^H NMR (400 MHz, DMSO‑*d*_6_) *δ* 13.05 (1H, br s), 10.61 (1H, s), 7.90 (1H, t, *J* = 8.6 Hz), 7.82 (1H, dd, *J* = 13.6, 2.0 Hz), 7.69 (1H, d, *J* = 2.0 Hz), 7.63 (1H, dd, *J* = 8.7, 2.0 Hz), 7.54 (1H, d, *J* = 2.1 Hz), 4.79 – 4.73 (1H, m), 4.63 – 4.57 (1H, m), 1.34 (6H, d, *J* = 6.0 Hz), 1.28 (6H, d, *J* = 6.2 Hz). m/z 410 [M+H]^+^ (ES^+^), 408 [M-H]^-^ (ES^-^).

**4-(3-Chloro-4,5-diisopropoxybenzamido)-2-methylbenzoic acid** (**44)** (103 mg, 48% for final step) (103 mg, 48% for final step) was prepared as a white solid in essentially the same manner as **42** except isopropyl bromide was used instead of cyclopentyl iodide and the reaction performed at 80 °C in *step (ix)* and that methyl 4-amino-2-methylbenzoate (**38,** R^1^ = Me) was used instead of methyl 4-aminobenzoate (**38,** R^1^ = H) in *step (x)*: ^1^H NMR (400 MHz, DMSO‑*d*_6_) *δ* 12.62 (1H, br s), 10.34 (1H, s), 7.87 (1H, d, *J* = 8.5 Hz), 7.73 (1H, dd, *J* = 8.5, 2.2 Hz), 7.68 (2H, d, *J* = 1.9 Hz), 7.54 (1H, d, *J* = 2.0 Hz), 4.78 – 4.72 (1H, m), 4.61 – 4.55 (1H, m), 2.54 (3H, s), 1.33 (6H, d, *J* = 6.0 Hz), 1.28 (6H, d, *J* = 6.2 Hz). m/z 404 [M-H]^-^ (ES^-^).

The compound **45** was similarly prepared as **41**:

**4-(3,4-Di-*tert*-butoxy-5-chlorobenzamido)-2-methylbenzoic acid** (**45)** (86 mg, 49% for final step) was prepared as a white solid in essentially the same manner as for **41**except that methyl 4-amino-2-methylbenzoate was used instead of methyl 4-aminobenzoate: ^1^H NMR (400 MHz, DMSO‑*d*_6_) *δ* 12.60 (1H, br s), 10.38 (1H, s), 7.89 – 7.82 (2H, m), 7.72 (1H, d, *J* = 2.2 Hz), 7.70 (1H, br s), 7.60 (1H, d, *J* = 2.2 Hz), 2.45 (3H, s), 1.41 (9H, s), 1.32 (9H, s). m/z 432 [M-H]^-^ (ES^-^).

The compounds **49** **–** **55,** **57** and **59** were similarly prepared as **56.**

**4-(3-Chloro-4-ethoxy-5-isopropoxybenzamido)benzoic acid (49)** (145 mg, 80% for final step)

was prepared as a white solid in essentially the same manner as **56** except that methyl 4-aminobenzoate (**38,** R^1^ = H) was used instead of methyl 4-amino-2-methylbenzoate (**38,** R^1^ = Me) in *step (viii)*: ^1^H NMR (400 MHz, DMSO‑*d*_6_) *δ* 12.77 (1H, br s), 10.46 (1H, s), 7.97 – 7.90 (2H, m), 7.90 –7.84 (2H, m), 7.68 (1H, d, *J* = 2.0 Hz,), 7.55 (1H, d, *J* = 2.0 Hz), 4.79 – 4.72 (1H, m), 4.11 (2H, q, *J* = 7.0 Hz), 1.35 – 1.28 (9 H, m). m/z 378 [M+H]^+^ (ES^+^), 376 [M-H]^-^ (ES).

**4-(3-Chloro-5-(cyclobutoxy)-4-ethoxybenzamido)benzoic acid (50)** (112 mg, 41% for final step) was prepared as a white solid in essentially the same manner as  **56** except that cyclobutyl bromide was used instead of isopropyl bromide in *step (iv)* and methyl 4-aminobenzoate (**38,** R^1^ = H) was used instead of methyl 4-amino-2-methylbenzoate (**38,** R^1^ = Me) in *step (viii)*: ^1^H NMR (400 MHz, DMSO‑*d*_6_) *δ* 12.75 (1H, br s), 10.46 (1H, s), 7.95**–**7.86 (4H, m), 7.71 (1H, d, *J* = 2.0 Hz), 7.37 (1H, d, *J* = 2.1 Hz), 4.89 – 4.82 (1H, m), 4.14 (2H, q, *J* = 7.0 Hz), 2.49**–**2.42 (2H, m), 2.19**–**2.01 (2H, m), 1.92**–**1.58 (2H, m), 1.33 (3H, t, *J* = 7.0 Hz). m/z 388 [M-H]^-^ (ES^-^).

**4-(3-Chloro-5-isopropoxy-4-methoxybenzamido)benzoic acid** (**51**) (47 mg, 56% for final step) was prepared as a white solid in essentially the same manner as **56** except that methyl iodide was used instead of iodoethane in *step (vi)* and methyl 4-aminobenzoate (**38,** R^1^ = H) was used instead of methyl 4-amino-2-methylbenzoate (**38,** R^1^ = Me) in *step (viii)*: ^1^H NMR (400 MHz, DMSO‑*d*_6_) *δ* 12.78 (1H, br s), 10.47 (1H, s), 7.94 (2H, d, *J* = 8.8 Hz), 7.88 (2H, d, *J* = 8.9 Hz), 7.69 (1H, d, *J* = 2.0 Hz), 7.58 (1H, d, *J* = 2.1 Hz), 4.80 – 4.74 (1H, m), 3.83 (3H, s), 1.33 (6H, d, *J* = 6.0 Hz). m/z 364 [M+H]^+^ (ES^+^), 362 [M-H]^-^ (ES^-^).

**4-(3-Chloro-5-ethoxy-4-isopropoxybenzamido)benzoic acid** (**52**) (10 mg, 4% for final step) was prepared as a white solid in essentially the same manner as **56** except that ethyl iodide at room temperature was used instead of isopropyl bromide in *step(iv)*, isopropyl bromide at 60 °C was used instead of iodoethane in *step (vi)* and methyl 4-aminobenzoate (**38,** R^1^ = H) was used instead of methyl 4-amino-2-methylbenzoate (**38,** R^1^ = Me) in *step (viii)*: ^1^H NMR (400 MHz, DMSO‑*d*_6_) *δ* 12.75 (1H, br s), 10.46 (1H, s), 7.98**–**7.83 (4H, m), 7.69 (1H, d, *J* = 2.0 Hz), 7.54 (1H, d, *J* = 2.1 Hz), 4.63 – 4.56 (1H, m), 4.17 (2H, q, *J* = 6.9 Hz), 1.40 (3H, t, *J* = 6.9 Hz), 1.28 (6H, d, *J* = 6.2 Hz). m/z 378 [M+H]^+^ (ES^+^), 376 [M-H]^-^ (ES^-^).

**4-(3-Chloro-5-isopropoxy-4-methoxybenzamido)-2-fluorobenzoic acid** (**53**) (116 mg, 76% for final step) was prepared as a white solid in essentially the same manner as **56** except that methyl iodide was used instead of ethyl iodide in *step (vi)* and that methyl 4-amino-2-fluorobenzoate (**38,** R^1^ = F) was used instead of methyl 4-amino-2-methylbenzoate (**38,** R^1^ = Me) in *step (viii)*: ^1^H NMR (400 MHz, DMSO‑*d*_6_) *δ* 13.03 (1H, br s), 10.60 (1H, s), 7.90 (1H, t, *J* = 8.6 Hz), 7.82 (1H, dd, *J* = 13.6, 2.0 Hz), 7.69 (1H, d, *J* = 2.0 Hz), 7.63 (1H, dd, *J* = 8.7, 2.0 Hz), 7.57 (1H, d, *J* = 2.0 Hz), 4.81 – 4.73 (1H, m), 3.85 (3H, s), 1.35 (6H, d, *J* = 6.0 Hz). m/z 380 [M-H]^-^ (ES^-^).

**4-(3-Chloro-4-ethoxy-5-isopropoxybenzamido)-2-fluorobenzoic acid** (**54**) (120 mg, 78% for final step) was prepared as a white solid in essentially the same manner as **56** except that methyl 4-amino-2-fluorobenzoate (**38,** R^1^ = F) was used instead of methyl 4-amino-2-methylbenzoate (**38,** R^1^ = Me) in *step (viii)*: ^1^H NMR (400 MHz, DMSO‑*d*_6_) *δ* 13.02 (1H, br s), 10.60 (1H, s), 7.90 (1H, t, *J* = 8.6 Hz), 7.82 (1H, dd, *J* = 13.7, 2.0 Hz), 7.69 (1H, d, *J* = 2.0 Hz), 7.63 (1H, dd, *J* = 8.7, 2.0 Hz), 7.55 (1H, d, *J* = 2.0 Hz), 4.80 – 4.73 (1H, m), 4.13 (2H, q, *J* = 7.0 Hz), 1.38-1.27 (9H, m). m/z 394 [M-H]^-^ (ES^-^).

**4-(3-Chloro-5-isopropoxy-4-methoxybenzamido)-2-methylbenzoic acid** **(55)** (94 mg, 57% for final step) was prepared as a white solid in essentially the same manner as **56** except that methyl iodide was used instead of ethyl iodide in *step(vi)*: ^1^H NMR (400 MHz, DMSO‑*d*_6_) *δ* 12.63 (1H, br s), 10.34 (1H, s), 7.86 (1H, d, *J* = 8.6 Hz), 7.75 **–** 7.63 (3H, m), 7.57 (1H, d, *J* = 2.1 Hz), 4.80 – 4.74 (1H, m), 3.83 (3H, s), 2.53 (3H, s), 1.34 (6H, d, *J* = 6.0 Hz). m/z 378 [M+H]^+^ (ES^+^), 376 [M-H]^-^ (ES^-^).

**4-(3-Chloro-5-ethoxy-4-isopropoxybenzamido)-2-methylbenzoic acid** (**57)** (129 mg, 56% for final step) was prepared as a white solid in essentially the same manner as **56** except that ethyl iodide at room temperature was used instead of isopropyl bromide in *step(iv)*, isopropyl bromide at 60 °C was used instead of methyl iodide in *step (vi)*: ^1^H NMR (400 MHz, DMSO‑*d*_6_) *δ* 12.68 (1H, s), 10.40 (1H, s), 7.92 (1H, d, *J* = 8.5 Hz), 7.81**–**7.69 (3H, m), 7.59 (1H, d, *J* = 2.1 Hz), 4.61 – 4.65 (1H, m), 4.21 (2H, q, *J* = 7.0 Hz), 2.58 (3H, s), 1.45 (3H, t, *J* = 6.9 Hz), 1.33 (6H, d, *J* = 6.2 Hz). m/z 390 [M-H]^-^ (ES^-^).

**4-(3-Chloro-5-(cyclopropylmethoxy)-4-ethoxybenzamido)-2-methylbenzoic acid** **(59)** (105 mg, 56% for final step) was prepared as a white solid in essentially the same manner as **56** except that bromomethylcyclopropane was used instead of isopropyl bromide in *step (iv)*: ^1^H NMR (400 MHz, DMSO‑*d*_6_) *δ* 12.65 (1H, br s), 10.35 (1H, s), 7.88 (1H, d, *J* = 8.6 Hz), 7.75**–**7.65 (3H, m), 7.55 (1H, d, *J* = 2.0 Hz), 4.17 (2H, q, *J* = 7.0 Hz), 4.00 (2H, d, *J* = 7.0 Hz), 2.54 (3H, s), 1.34 (3H, t, *J* = 7.0 Hz), 1.31**–**1.25 (1H, m), 0.65**–**0.58 (2H, m), 0.43**–**0.35 (2H, m). m/z 402 [M-H]^-^ (ES^-^).

The compound **58** was similarly prepared as **18**.

**4-(3-Chloro-4-ethoxy-5-methoxybenzamido)-2-methylbenzoic acid** (**58)** (97 mg, 52% for final step) was prepared as a white solid in essentially the same manner as **18** except that 3-chloro-4-ethoxy-5-methoxybenzoic acid (prepared in 3 steps from 3-chloro-4-hydroxy-5-methoxybenzoic acid **35** by sequential treatment with chlorotrimethylsilane and methanol, ethyl iodide and base and then lithium hydroxide) was used instead of 3,5-dichloro-4-ethoxybenzoic acid in *step(iii)*: ^1^H NMR (400 MHz, DMSO‑*d*_6_) *δ* 12.65 (1H, br s), 10.39 (1H, s), 7.88 (1H, d, *J* = 8.6 Hz), 7.76**–**7.66 (3H, m), 7.58 (1H, d, *J* = 2.0 Hz), 4.10 (2H, q, *J* = 7.0 Hz), 3.93 (3H, s), 2.55 (3H, s), 1.32 (3H, t, *J* = 7.0 Hz). m/z 364 [M+H]^+^ (ES^+^), 362 [M-H]^-^ (ES^-^).

**Biological assays.**

**Production of the biotin-RAR-ligand binding domain.**

Biotin-tagged RAR*α* ligand binding domain was produced and purified using the PinPoint™ Xa Protein Purification System (Promega). The mouse RAR*α* sequence encoding the ligand binding domain of the protein was amplified by PCR using primers 5AX 5’-GCT AAG CTT TCC AAG GAG TCG GTG CGA-3’ and 3AC 5’-CAG AGA TCT TCA TGG GGA TTG GGT GGC-3’, and ligated into the MCS of pXa3 using HindIII and BglII. The resulting pXa3-RAR*α* lbd vector was used to transform chemically competent JM109 strain E. coli (Promega). Bacteria were grown overnight at 37 °C in Luria broth with 100 mg/mL of ampicillin and 2 *μ*M biotin (Sigma), this confluent culture was used to inoculate a 1 l flask of LB-Amp with biotin, after 1 h of growth, optical density was tracked and the bacteria were cooled upon reaching an OD-600nm of 0.6. IPTG was added at a final concentration of 100 *μ*M to induce production of the biotin-tagged protein and bacteria were shaken at 26 °C for 2 h and then harvested by centrifugation. The bacterial pellet was opened by sonification and the protein was purified using the SoftLink™ Soft Release Avidin Resin (Promega) as per manufacturer’s instructions. The final eluted protein was stored at -80 °C in a stabilizing solution (50mM Tris (pH 8,0), 50mM NaCl, 4 mM DTT, 0,1% Triton, 2mM EDTA, 25% glycerol) with a cocktail of protease inhibitors. Excess biotin was removed by dialysis using Slide-a-Lyzer cassettes (Pierce), as per manufacturer’s instructions. Protein concentration was determined using the Bradford assay (Pierce).

**The FlashPlate® Scintillation Proximity Binding Assay.**

The Plating Buffer (pH 7.5) consisted of 25mM NaH2 PO4, 0.5mM MgCl2, 1mM DTT, 1mM EDTA, 5mM CHAPS, 10% Glycerol, 0.002% Tween -80, 0.5ng/*μ*l biotinylated RAR protein and 1x Protease Inhibitors (Calbiochem). The assay Buffer (pH 7.5), 25mM NaH2 PO4 0.5mM MgCl2 1mM DTT 1mM EDTA 5mM CHAPS, 10% Glycerol, 0.002% Tween -80, (2.0nM [3H]-RA), 1x Protease Inhibitors (optional). The Wash Buffer (pH 7.5), consisted of 25mM NaH2 PO4, 0.5mM MgCl2, 1mM DTT, 1mM EDTA, 5mM CHAPS, 10% Glycerol. Retinoids were Prepared in DMSO at 40x final concentration.

Streptavidin FlashPlates® (Perkin Elmer, Waltham, Mass., USA) were coated overnight at 4 °C with 15 ng/well of biotinylated RAR*α* ligand binding domain, in 200 mL scintillation proximity assay (SPA) plating buffer per well. Wells were washed with 3 x 300*μ*L ice-cold SPA wash buffer immediately prior to use. Radioligand was prepared by adding 2*μ*L [3H]ATRA per 20mL SPA plating buffer and 195*μ*L of this mixture added per well. Compounds were serially diluted in DMSO from 3*μ*M to 10nM in half log unit steps and duplicate 5*μ*L aliquots added to the FlashPlate. Duplicate 5*μ*L aliquots of a serially diluted ATRA standard curve were also added and the FlashPlate sealed, wrapped in foil and incubated with gentle shaking. After an overnight incubation, plates were read by a MicroBeta® TriLux luminescence counter (Perkin Elmer). Results were plotted and analyzed by GraphPad Prism®.

**Transient Cell Transfections for Transactivation Studies.**

The mouse RAR sequences encoding the ligand binding domain of the protein were amplified by PCR using the following primers. RAR*α*, forward Ecor I, gct gga attc atg gcc agc aat agc agt, reverse, Bgl II cag agatct tca tgg gga ttg ggt ggc; RAR*β*, forward HpaI, gct gttaac atg ttt gac tgt atg gat, reverse, BamH I, caa ggatc tca ctg cag cag tgg tga; RAR*γ*, forward, Ecor I, gct gga attc atg gcc acc aat aag gag, reverse Bgl II, cag agatct tca ggg ccc ctg gtc agg. The fragments were cloned into the overexpression vector PGS5 (stratagene). The resulting PGS5-RAR lbd vector was used to transform chemically competent JM109 strain E. coli (Promega). Bacteria were grown overnight at 37 °C in Luria broth with 100 *μ*g/mL of ampicillin. DNA was isolated using Promega maxiprep kits. The day before transfection, COS-7 cells are seeded on 24-well culture plates at a density of 5x10^4^ cells per well so that they are 50–70% confluent on the day of transfection. Cells were cotransfected, with 0.1 *µ*g of reporter vector pRARE-tk-Luc that allows the expression of the reporter gene firefly luciferase, 0.05 *µ*g of pSG5-RAR*α*, pSG5-RAR*β*, or pSG5-RAR*γ* expression vectors and 0.02 *µ*g of the pRLnull vector that encodes the Renilla luciferase (Promega) as an internal control to normalize for variations in transfection efficiency. The total DNA (0.17 *µ*g /well) was diluted with 25 *μ*L of DMEM and mixed with 2 *μ*L of Plus reagent and incubated for 15 min at room temperature. LipofectAMINE (0.6 *μ*L) with 25 *μ*L of DMEM is added and incubated for 15 min at room temperature. The cells are washed twice with DMEM to eliminate serum traces, and 200 *μ*L of serum-free DMEM was added per well. The mixture was added to the cells and incubated at 37 °C. After 3 h of incubation, the culture volume was increased to 1 mL per well with DMEM supplemented 10% serum (Invitrogen). Twenty-four hrs after transfection, the cells were washed and then treated with the appropriate retinoid in serum-free medium for 12hrs. The cells were then washed twice with cold phosphate-buffered saline (PBS) and lysed with 200 *μ*L*α* per well of passive lysis buffer (Promega) and incubated for 15 min at room temperature.

**Dual Luciferase Assays**

Firefly luciferase and Renilla luciferase activities were determined on 10 *µ*l of lysate using the Dual luciferase assay system kit (Promega). The light intensity was determined with a luminometer after injecting 50 *µ*l of LAR II reagent (firefly luciferase substrate), and 50 *μ*L of Stop and Glo reagent (Renilla luciferase substrate), successively. All the experiments were carried out in triplicate, and the Firefly luciferase activity is normalized with the Renilla activity (efficiency of transfection) and with the protein content.

**Data Analysis**

Percent maximal response data, or [^3^H]ATRA competition from RARs by metabolites, were fitted to a sigmoidal equation using GraphPad Prism version 3.0 software (San Diego, CA). Binding data is presented as IC_50_ values, i.e. the concentration of unlabeled compound required to prevent 50% of the radiolabeled ligand from binding to the receptor. Data for the transactivation studies are presented as EC_50_ values, which represent the effective concentration for half-maximal luciferase activity. Both the binding and transactivation assays are highly reproducible and are used as a standard for analysis of retinoids.

**Invitrogen Transactivation RAR assays** (Life Technologies' SelectScreen® Profiling Service: 10-point Titration Agonist Results). GeneBLAzer® technology uses a mammalian-optimized Beta-lactamase reporter gene (*bla*) combined with an FRET-enabled substrate to provide reliable and sensitive detection in intact cells.

The GeneBLAzer® RAR alpha DA (Division Arrested) and RAR alpha-UAS-*bla* HEK 293T cells contain the ligand-binding domain (LBD) of the human retinoic acid receptor alpha fused to the DNA-binding domain of GAL4 stably integrated in the GeneBLAzer®UAS-bla HEK293T cell line. GeneBLAzer®UAS-bla HEK 293T cells (catalog#K1104) stably express a beta-lactamase reporter gene under the transcriptional control of a 7 x Upstream Activator Sequence (UAS). Transcription from the 7 x UAS is activated by the binding of the GAL4 transcription factor DNA-binding-domain (DBD). The GAL4-DBD is expressed as a fusion protein with the ligand binding domain (LBD) of RAR alpha. When an agonist binds to the LBD of the GAL4(DBD)-RAR alpha(LBD) fusion protein it translocates to the nucleus where it binds to the 7 x UAS inducing transcription of beta-lactamase. Division Arrested (DA) cells are available in an Assay Kit (which includes cells and sufficient substrate to analyze 1 x 384-well plate). DA cells are irreversibly division arrested using a low-dose treatment of Mitomycin-C, and have no apparent toxicity or change in cellular signal transduction.

**RAR-alpha - Agonist Screen**

RAR-alpha-UAS-bla HEK 293T cells are thawed and resuspended in Assay Media (DMEM phenol red free, 2% CD-treated FBS, 0.1 mM NEAA, 1 mM Sodium Pyruvate, 100U/mL/100g/mL Pen/Strep) to a concentration of 312,500 cells/mL. 4 *μ*L of a 10X serial dilution of ATRA (control agonist starting concentration, 10 nM) or compounds are added to appropriate wells of a 384-well TC-Treated assay plate. 32 *μ*L of cell suspension (10,000 cells) is added to each well. 4 *μ*L of Assay Media is added to all wells to bring the final assay volume to 40 *μ*L. The plate is incubated for 16-24 hours at 37 °C /5% CO_2_ in a humidified incubator. 8 *μ*L of 1 *μ*M Substrate Loading Solution is added to each well and the plate is incubated for 2 hours at room temperature. The plate is read on a fluorescence plate reader.

Similarly done for the corresponding **RAR beta** and **RAR gamma receptors**

<https://www.thermofisher.com/uk/en/home/products-and-services/services/custom-services/screening-and-profiling-services/selectscreen-profiling-service/selectscreen-cell-based-nuclear-receptor-profiling-services.html>

**In Vivo Rat Pharmacokinetics and Oral Bioavailability**

The study was conducted by CXR Biosciences Ltd, James Lindsay Place, Dundee Technopole, Dundee DD 5JJ

**In Vivo Mouse Pharmacokinetics and Oral Bioavailability.**

In the in vivo study (study number S-BF-118), **56** was administered in solution (2% DMSO in 0.05M phosphate buffered saline, pH 7.4) to mice at a dose of 1mg/kg for both routes in dose volumes of 5 mL/kg (IV) and 10 mL/kg (PO). Blood was collected into tubes containing 3.8% w/v tri-sodium citrate as anticoagulant and to help stabilise any glucuronide metabolites of test compounds that may be present in the samples. Blood was centrifuged to yield plasma for quantification of parent compound using UPLC-MS/MS. Compound **56** was administered to groups of twenty seven mice (three mice per time-point at 0.08, 0,25, 0,5, 1, 2, 4, 8, 12, 24 hrs). Terminal blood samples (approximately 300 μL) were subsequently taken by cardiac puncture under CO_2_ terminal anaesthesia. Once collected, blood samples were transferred to tubes containing 28 μL of citrate as anti-coagulant and held on ice, for <30 minutes, before centrifugation to generate plasma. Plasma samples aliquots (50 μL) were treated with four volumes of acetonitrile, containing diclofenac as analytical internal standard. Precipitated proteins were removed by centrifugation and the supernatant was analysed by UPLC-MS/MS. Pharmacokinetic parameters were determined by non-compartmental analysis using the software package PK Solutions 2.0 from Summit Research Services. The study was conducted by BioFocus, Chesterford Research Park, Little Chesterford, Essex, CB10 1X [www.biofocus.com](http://www.cylex-uk.co.uk/reviews/viewcompanywebsite.aspx?firmaName=bio+focus&companyId=15721885)

**In Vivo Dog Pharmacokinetics and Oral Bioavailability.**

Three male Beagle dogs were administered **56** by oral gavage at a dose level of 1 mg/kg or

intravenous (bolus) injection at a dose level of 0.2 mg/kg. Oral and intravenous formulations produced clear solutions in 2% DMSO : 98% 0.05M phosphate buffered saline (pH 7.4). Intravenous doses were administered by bolus injection into a cephalic vein at a dose volume of 1 mL/kg. Oral doses were administered by gastric intubation at a dose volume of 5 mL/kg. Blood samples were taken at the following time-points: pre-dose, 15 and 30 minutes and 1, 1.5, 2, 3, 4, 6, 8, 12 and 24 hours post-dose, following oral administration and pre-dose, 5, 15 and 30 minutes and 1, 1.5, 2, 3, 4, 6, 8, 12 and 24 hours post dose, following IV administration. Plasma concentrations of **56** were measured by a qualified liquid chromatographic - tandem mass spectrometric (LC-MS/MS). Pharmacokinetic parameters were calculated using the computer program Phoenix WinNonlin version 6.3 (Pharsight Corporation, USA). The study was conducted by Huntingdon Life Sciences, Huntingdon Research Centre, Woolley Road, Alconbury, Huntingdon, Cambridgeshire, PE28 4HS, UK.

**ADME assays**

Cyprotex Discovery Ltd,15 Beech Lane, Macclesfield, Cheshire SK10 2DR United Kingdom [**http://www.cyprotex.com/services**](http://www.cyprotex.com/services)

**LogD**: Cyprotex's Log D 7.4 assay uses the octanol/buffer shake flask method for determining lipophilicity. Briefly, octanol (pre-saturated with buffer) is added to the test compound and sonicated. Buffer at pH 7.4 (presaturated with octanol) is then added to the octanol. A ratio of 2:1 v/v buffer to octanol is prepared. The system is mixed to allow distribution of the compound between the two phases. After separation, the compound is quantified in the aqueous phase and octanol phase by LC-MS/MS and the following equation is used to calculate the Log D 7.4.

**Log D** = Log (C_oct_ / C_aq_ ). Where: Coct = Concentration in octanol sample (corrected for dilution) and Caq = Concentration in aqueous sample (corrected for dilution)

**Intrinsic clearance Cl_int_**: (Cloe Screen Microsomal Stability Fact Sheet)

**Human plasma protein binding**

Equilibrium dialysis is used to determine the extent of binding of a compound to plasma proteins. A semi-permeable membrane separates a protein-containing compartment from a protein-free compartment. The system is allowed to equilibrate at 37 °C. The test compound present in each compartment is quantified by LC-MS/MS. The extent of binding is reported as a fraction unbound (fu) value which is calculated as detailed below;

fu = 1 – (PC - PF /PC). PC = Test compound concentration in protein-containing compartment. PF = Test compound concentration in the protein-free compartment.

**Cyp 450 liability:** Cloe Screen Cytochrome P450 Inhibition Fact Sheet

The five main cytochrome P450 isoforms (CYP1A, CYP2C9, CYP2C19, CYP2D6 and CYP3A4) were investigated in the P450 inhibition assay. Isoform-specific substrates were incubated individually with human liver microsomes and a range of test compound concentrations (0.05 - 25*µ*M). At the end of the incubation, the formation of the metabolite was monitored by LC-MS/MS (or fluorescence in the case of CYP1A) at each of the test compound concentrations. A decrease in the formation of the metabolites compared to vehicle control was used to calculate an IC_50_ value (test compound concentration which produces 50% inhibition).

**Stability and intrinsic clearance in hepatocytes.**

Incubations of the test compound **56** (1 μM and 30 μM initial concentration, n=2) were carried out with pooled cryopreserved hepatocytes (Celsis IVT) at cell densities of 0.5 million cells/mL for human (batch TVP), rat (batch DBL), cynomolgus monkey (batch MJH) and dog (batch KLI) and 0.25 million cells/mL for mouse (batch XYG). The incubations were performed at 37 °C with 100 μL samples taken from the incubation at 0, 30, 60, 90, 120 and 180 minutes for **56** and the assay control compounds (1 μM), metoprolol (mouse, rat, Cynomolgus macaque and human) and quinidine (dog). The control compounds midazolam, testosterone, 4-methylumbelliferone were sampled out to either 120 or 180 minutes depending on species. Reactions were terminated by addition of 100 μL of acetonitrile containing carbamazepine as the analytical internal standard. Samples were centrifuged and the supernatant fractions analysed by LC-MS/MS. The instrument responses (peak heights) were referenced to the zero time-point samples (as 100%) in order to determine the percentage of compound remaining. Ln plots of the % remaining, for each compound, were used to determine the half-life for the hepatocyte incubations. Half-life values were calculated from the relationship T_1/2_ (min) = -0.693/λ where λ was the slope of the Ln concentration vs time curve. Standard compounds testosterone, midazolam and 4-methylumbelliferone are included in the assay design. These compounds give an indication of the metabolic capacity of the cryopreserved preparations for both Phase I and Phase II reactions. In vitro intrinsic clearance (Cl_int_) as μL/min/million cells was calculated by applying the following formula to the half-life values: Cl_int_ in vitro (μL/min/million cells) = [0.693 x 1/T½ (min)] x [1/million cells per mL]*1000. The study was conducted by BioFocus, Chesterford Research Park, Little Chesterford, Essex, CB10 1X [www.biofocus.com](http://www.cylex-uk.co.uk/reviews/viewcompanywebsite.aspx?firmaName=bio+focus&companyId=15721885)

**Cellular Toxicity assays** (Cyprotex)

**High-Content Cell Toxicity Screen in HEPG2 cells:**

Effect on the cell or mitochondrial viability markers in HepG2 cells were tested for compounds up to 50 *μ*M. HepG2 cells were plated on 96-well tissue culture treated black walled clear bottomed polystyrene plates at 0:3 _ 104 cells in 100_L per well. After 24 hr the cells were dosed with the test compound at a range of concentrations (see Assay Summary for details). At the end of the incubation period, the cells were loaded with the relevant dye/antibody for each cell health marker. The plates were then scanned using an automated fluorescent cellular imager, ArrayScanr VTI (Thermo Scientific Cellomics).

**Cytotoxicity Assay:** COS-7 cells were plated out on 96 well plates at a density of 8000 cells per well. Twenty-four hours after plating retinoids at 50, 10 and 2 x EC_50_ values were added to the wells. After two days an MTT assay (Promega) was carried out according to the manufacturer’s instructions

**Genetic Toxicity**

**Cerep**, Le Bois l’Evêque, B.P. 30001, 86 600 Celle l’Evescault, France. Tel: +33 (0)5 49 89 30 00. <http://www.cerep.fr/cerep/users/pages/ProductsServices/InVitro.asp>

**Genotoxicity Profile: Cerep**.

This assay is comprised of three tests that were used to evaluate the properties of compound **56**.

**Bacterial Cytotoxicity**

The bacterial cytotoxicity of compound **56** was evaluated on 3 bacterial strains of Salmonella Typhimurium (TA98, TA100, and TA1535) with 8 concentrations of compound **56** ranging from 6.3E-07M to 1.0E-04M, according to a published protocol.**^1^** The reference compound was mitomycin C. The results for cytotoxicity are expressed as percent of control growth (OD_650_). A compound with growth less than 60% of control is flagged and considered to be cytotoxic.

**Ames test**

Compound **56** was evaluated in the Ames test on the same three Salmonella Typhimurium bacterial strains in the presence or absence of rat liver S9, Compound **56** was evaluated at concentrations ranging from 5.0E-06M up to 1.0E-04M, according to a published protocol.^1^ The reference compounds in these assays were aminoanthracene, mitomycin C, quercetin, and streptozotocin. For the first two assays, photometry is used for detection.

**In vitro Micronucleus test**

Compound **56** was evaluated in the in vitro micronucleus assay conducted in CHO-K1 cells, in the presence or absence of rat liver S9, according to a published protocol.^2^ Compound **56** was evaluated at concentrations ranging from 3.1E-05M up to 5.0E-04M in the presence of S9 serum where the reference product was cyclophosphamide, and at concentrations ranging from 1.6E-05M up to 5.0E-04M in the absence of S9 serum where the reference product was mitomycin. Fluorescent imaging was used to obtain the results in the micronucleus test.

**In Vitro Pharmacology: Binding and Enzyme Assays: Cerep**.

The compound **56** was tested in two independent experiments at all targets using a test concentration of 10 μM. The CEREP selectivity screen was performed on the following targets:

A_1_, A_2A_, A_3_, α_1_, α_2_, β_1_, β_2_, AT_1_, AT_2_, BZD central, BZD periph, BB, B_2_, CGRP, CB_1_, CCK_1_, CCK_2_, D_1_, D_2S_, D_3_, D_4.4_, D_5_, ET_A_, ET_B_, GABA, GAL_1_, GAL_2_, PDGF, CXCR2, CCR1, TNF- α, H_1_, H_2_, MC_4_, MT_1_, M_1_, M_2_, M_3_, M_4_, M_5_, NK_1_, NK_2_, NK_3_,Y_1_, Y_2_, NTS_1_(NT_1_), δ_2_, κ, μ, NOP(ORL1) , PAC1(PACAP), PPARγ, PCP, EP_2_, EP_4_, IP(PGI_2_,), P2X, P2Y,5-HT_1A_, 5-HT_1B_, 5-HT_2A_, 5-HT_2B_, 5-HT_2C_, 5-HT_3_, 5-HT_5A_, 5-HT_6_, 5-HT_7_, σ, sst, GR, ER, VPAC_1_(VIP_1_), V_1a_, Ca^2+^ channel (L), K_V_ channel, K_V_ channel, SK_Ca_ channel, Na^+^ channel, Cl^-^ channel, norepinephrine transporter. dopamine transporter, 5-HT transporter.

sPLA_2_, COX_1_, COX_2_, 5-lipoxygenase, 12-lipoxygenase, inducible NOS, PDE2A_1_, PDE3A, PDE4D_2_, PDE5, PDE6, ACE, ACE-2, BACE-2, ECE-1, elastase, caspase-3, caspase-8, cathepsin D, cathepsin L, neutral endopeptidase, MMP-1, MMP-3, MMP-9, tryptase, TACE, phosphatase 1B, phosphatase CDC25A, Scr kinase, acetylcholinesterase, MAO-A, MAO-B, CENP-E, Eg5, HDAC3, HDAC4, HMG-CoA reductase, HDAC11, surtuin-1, surtuin-2.

All results showed inhibition (or stimulation) ≤25% and were not considered significant. The highest inhibition of 25% was found for the 5HT_2B_ site.

**hERG Binding Assay**.

Interaction with the hERG channel was assessed by displacement of the radioligand [^3^H]-astemizole as described by Chiu et al.^3^

**Virtual Screening and Molecular Modelling**.

**Cresset Biomolecular Discovery**, Broadwater Road, Welwyn Garden City, Hertfordshire AL7 3AX. Tel: 01707356120. http://www.cresset-bmd.com

# Virtual Screening Project for King’s RARα Project

The objective was to find novel small molecule mimetics of selective RARα agonists using Cresset’s FieldScreen™ technology (Cresset Project CBMD036). This work was greatly facilitated by the availability of RAR*α*, protein X-ray structure in the PDB database 1DKF.pdb. RAR-RXR-*α* containing the antagonist BMS195614 (**1**) in the RAR*α* active site.

**Cresset Modelling**

The X-ray data of RAR*α* complexed with its antagonist BMS195614 (1DFK.pdb) was firstly reduced down to those residues specifically involved with the active site (Ser214-Asn416), given 1/8^th^ full charge at all groups likely to be ionised at pH7, allocated XEDs according to the rules of the XED force field ^4^ and fully minimised with no electrostatic cutoff and 15Ang. vdW cutoff to an exit level of 0.08 rms deviation on all atoms.

1. ***Substituting ligands into the active site***

Taking the reduced minimized X-ray structure, now called the ‘working protein', AM580 (**2**) was substituted into the same space as BMS195614 (**1**) over corresponding atoms and the whole ensemble was minimized to 0.1rms exit level. This procedure was repeated with AGN193836 (**3**) replacing AM580. Figures 3 and 4 show the movements of ligand and surrounding protein before and after minimization.

Figure 4 clearly shows that the acid group of AGN193836 has been twisted out of the plane by the two adjacent fluorine atoms. This effect may be important in increasing binding in the RAR active site and may also have bearing on the selectivity indices. This observation coupled with a number of other revelations; namely that:

1. Selectivity seems to be associated with an H-bond uniquely associated with the RAR*α* S232 residue
2. Docking suggests that the amide linkage may bind either way round
3. There seems to be room within the hydrophobic pocket of the active site to allow the smaller groups also to bind either way round.

prompted a more extensive modeling study into the binding modes and poses that these RAR ligands could adopt.

**Figure 3.** C*α* overlay of the minimized protein with BMS195614 and the minimized protein after the antagonist was substituted with AM580. RMSD=0.27.

**Figure 4** C*α* overlay of the minimized protein with AM580 and the minimized protein after AGN193836 was substituted for AM580 shown in Figure 3. RMSD=0.06.

1. ***In search of the most efficacious bioactive conformation***

Given that – AM580 and AGN193836 – might not be binding in the same pose as the antagonist BMS195614 used in the X-ray, a program of ligand minimization was undertaken. This involved substituting these ligands into the active site as already described and calculating their binding energies after full minimization. Binding energy is the sum of the electrostatic attraction and the van der Waals dispersion energy which together account for the energy of binding of two fragments by non-bonding forces. In this case, the ‘Binding Energy’ is calculated between the ligand and the protein.


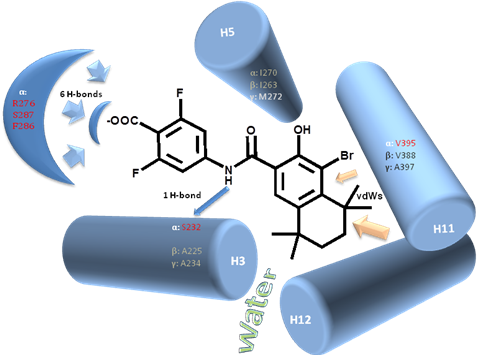
Table 1 reports the binding energies of the ligands in various conformations (in kcal/mol). The overall binding energy is accompanied by it's electrostatic (coulombic) and dispersive (vdW) contributions (grayed).

With reference to the picture opposite, where S232 is below on Helix 3 and the acid of each ligand is to the left, each conformer in Table 1 is coded either ‘CO up or down' and the hydrophobic part of the ligand on the right is coded ‘HY up or down'. Note that the X-rayed antagonist pose is therefore defined as ‘HYd' and ‘COu'. And the picture opposite is of AGN193836 ‘HYd COu'.

Firstly, these binding energy numbers are approximate. They take no account of peripheral issues such as entropy and solvation changes and, as such, they cannot be associated with biological activity. If, however, the peripheral factors are much the same for all ligand, such a correlation could be made. Secondly, the binding energy numbers have an estimated calculation error of 2 kcal/mol so that -83.2 may not be different from -84.4.

| **Protein + Ligand** | **Ligand Structure** | **Binding**  **Energy** | **Coulomb**  **Energy** | **vdW**  **Energy** |
| --- | --- | --- | --- | --- |
| **RAR*α*** |  |  |  |  |
| *α*RAR+BMS195614_HYd-COu  X-ray minimized  Antagonist |  | **-115.6** | -16.0 | -99.6 |
| *α*RAR+AM580_HYd_COu |  | **-83.2** | -6.0 | -77.2 |
| *α*RAR+AM580_HYu_COu |  | **-85.4** | -8.1 | -77.3 |
| *α*RAR+AM580_HYu_COd |  | **-87.8** | -12.1 | -75.7 |
| *α*RAR+AM580_HYd_COd |  | **-92.2** | -15.0 | -77.2 |
| *α*RAR+AGN193836_HYd_COu |  | **-94.0** | -10.5 | -83.5 |
| *α*RAR+AGN193836_HYu_COu |  | **-92.9** | -10.4 | -82.5 |
| *α*RAR+AGN193836_HYd_COd |  | **-92.4** | -14.1 | -78.3 |
| *α*RAR+AGN193836_HYu_COd |  | **-93.6** | -13.5 | -80.1 |

**Table 1**. Binding energies of ligands in various conformations (in kcal/mol). The overall binding energy is accompanied by it's electrostatic (coulombic) and dispersive (vdW) contributions (grayed). See text for the codes defining each ligand conformation.

Table 1 suggests some interesting conclusions:

- The antagonist, BMS195614, binds more strongly than any of the agonists with most of its binding associated with the vdW dispersion, probably of the extended arm. This is intuitively acceptable.
- Generally, AM580 can bind in four different ways although the binding energies might suggest a preference for the amide carbonyl to form an H-bond with S232.
- AGN193836 shows no such preference and binds happily in all four poses. This is entropically favorable. The binding energy itself suggests that this ligand is the most active of all the agonists.

These results indicate that the preferred conformations for the chosen compounds should be all

four conformers:

**1** AM580-COup-HYdown **2** AGN193836-COup-HYdown

**3** AM580-COdown-HYdown **4** AGN193836-COdown-HYdown

As a first pass, only **1** and **2** were used to search the FieldScreen database.

Molecular fields were plotted around each molecule, based on its electronic makeup, surface, and hydrophobic properties. These fields were distilled down to their local extrema and the resulting field points (positive red points, negative blue points, surface yellow points and hydrophobic orange points) reflecting how the molecule interacts with its environment.**^4^** These are shown below with their fields ready for seeding a search the FieldScreen database.

- **1 (AM580-COup-HYdown) with fields used as seed 1 in the FieldScreen search**
- **2 (AGN193836-COup-HYdown) with fields used as seed 2 in the FieldScreen search**

These unique molecular field patterns were used to search Cresset's database of 2.5M commercially available molecules and the results ranked in similarity to the initial bioactive conformations.**^4, 5^** This methodology identified 3000 commercially available compounds as possible hit compounds. The 200 compounds that had the highest field overlays, Lipinski likeness, and synthetic tractability, were purchased. These were tested in transactivation assays at the RAR*α*, *β* and *γ* receptors.

**References**.

(1) Maron, D.M.; Ames, B.N. Revised methods of the salmonella mutagenicity test. *Mutat. Res*. **1983**, *113*, 173**–**215.

(2) Diaz, D.; Scott, A.; Carmichael, P.; Shi, W.; Costales, C. Evaluation of an automated in vitro micronucleus assay in CHO-K1 cells. *Mutat. Res*, **2007**, *630*, 1**–**13.

(3) Chiu, P.J.S.; Marcoe, K.F.; Bounds, S.E.; Lin, C.-H.; Feng, J.- J.; Lin, A.; Cheng, F.-C.; Crumb, W.J.; Mitchell, R. Validation of a [^3^H]astemizole binding assay in HEK293 cells expressing HERG K^+^ channels. *J. Pharmacol. Sci*. **2004**, *95*, 311**–**319.

(4) Cheeseright, T.; Mackey, M.; Rose, S.; Vinter, A. Molecular field technology applied to virtual screening and finding the bioactive conformation. [*Expert Opin. Drug Discov.*](https://www.ncbi.nlm.nih.gov/pubmed/23496041) **2007**, *2*, 131-144.

(5) Low, C. M. R.; Vinter, J. G. Rationalizing the Actives of Diverse Cholecystokinin 2 Receptor Antagonists Using Molecular Field Points *J. Med. Chem.* **2008**, 51, 565-573
